# Supplementary material for: Clinical Relevance of a 16-Gene Pharmacogenetic Panel Test for Medication Management in a Cohort of 135 Patients
Source: J Clin Med. 2021 Jul 21;10(15):3200. doi: 10.3390/jcm10153200 (PMC8347064; doi:10.3390/jcm10153200)
Supplement: Supplementary file 1 [file jcm-10-03200-s001.zip › jcm-1293788-supplementary.pdf]

Clinical Relevance of a 16-Gene Pharmacogenetic Panel Test for Medication Management in a Cohort of 135 Patients

David Niedrig[1,2], Ali Rahmany[1,3], Kai Heib[4], Karl-Dietrich Hatz[4], Katja Ludin[5], Andrea M. Burden[3], Markus Béchir[6], Andreas Serra[7], Stefan Russmann[1,3,7,*]

1 drugsafety.ch; Zurich, Switzerland
2 Hospital Pharmacy, Clinic Hirslanden Zurich; Zurich Switzerland
3 Swiss Federal Institute of Technology Zurich (ETHZ); Zurich, Switzerland
4 INTLAB AG; Uetikon am See, Switzerland
5 Labor Risch, Molecular Genetics; Berne, Switzerland
6 Center for Internal Medicine, Clinic Hirslanden Aarau; Aarau, Switzerland
7 Institute of Internal Medicine and Nephrology, Clinic Hirslanden Zurich; Zurich, Switzerland

* Correspondence: rustefan@ethz.ch; Tel.: +41 (0)44 221 1003

Supplementary Tables, Figures and Other Documents

Figure S1: Example of credit-card sized pharmacogenomic profile issued to patients

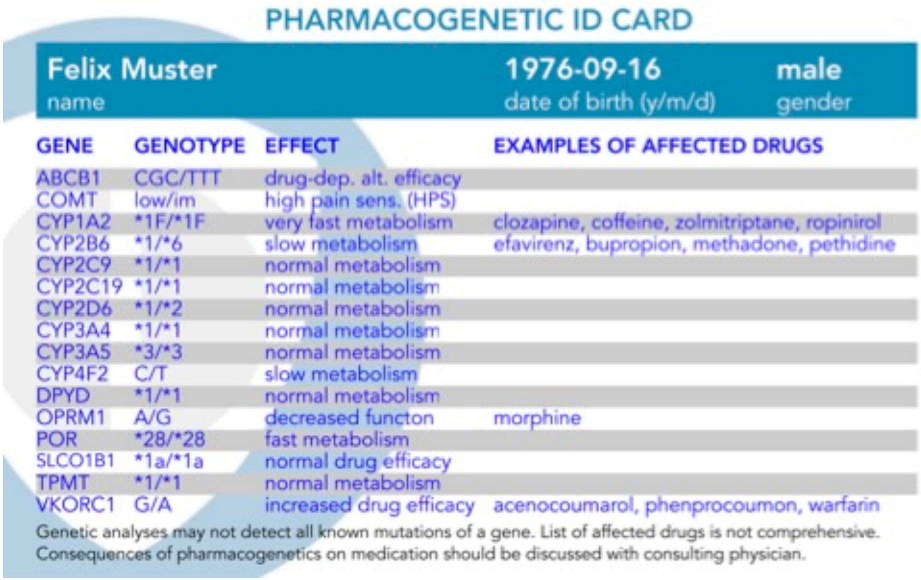

PHARMACOGENETIC ID CARD

Felix Muster (name) — 1976-09-16 (date of birth (y/m/d)) — male (gender)

GENE	GENOTYPE	EFFECT	EXAMPLES OF AFFECTED DRUGS
ABCB1	CGC/TTT	drug-dep. alt. efficacy	
COMT	low/im	high pain sens. (HPS)	
CYP1A2	*1F/*1F	very fast metabolism	clozapine, coffeine, zolmitriptane, ropinirol
CYP2B6	*1/*6	slow metabolism	efavirenz, bupropion, methadone, pethidine
CYP2C9	*1/*1	normal metabolism	
CYP2C19	*1/*1	normal metabolism	
CYP2D6	*1/*2	normal metabolism	
CYP3A4	*1/*1	normal metabolism	
CYP3A5	*3/*3	normal metabolism	
CYP4F2	C/T	slow metabolism	
DPYD	*1/*1	normal metabolism	
OPRM1	A/G	decreased functon	morphine
POR	*28/*28	fast metabolism	
SLCO1B1	*1a/*1a	normal drug efficacy	
TPMT	*1/*1	normal metabolism	
VKORC1	G/A	increased drug efficacy	acenocoumarol, phenprocoumon, warfarin

Genetic analyses may not detect all known mutations of a gene. List of affected drugs is not comprehensive.
Consequences of pharmacogenetics on medication should be discussed with consulting physician.

Table S1: SNPs analyzed by the 16-gene panel test

Gene	Allele	rs number
ABCB1	*Haplotypes 1236-2677-3435*	*rs1045642*
ABCB1		*rs1128503*
ABCB1		*rs2032582*
COMT	*Haplotypes 6269-4633-4818-4680*	*rs4633*
COMT		*rs4680*
COMT		*rs4818*
COMT		*rs6269*
CYP1A2	**1C*	*rs2069514*
CYP1A2	**1F*	*rs762551*
CYP1A2	**1K*	*rs12720461*
CYP1A2	**7*	*rs56107638*
CYP1A2	**11*	*rs72547513*
CYP2B6	**6*	*rs3745274*
CYP2B6	**18*	*rs28399499*
CYP2C19	**2*	*rs4244285*
CYP2C19	**3*	*rs4986893*
CYP2C19	**4*	*rs28399504*
CYP2C19	**5*	*rs56337013*
CYP2C19	**6*	*rs72552267*
CYP2C19	**7*	*rs72558186*
CYP2C19	**8*	*rs41291556*
CYP2C19	**17*	*rs12248560*
CYP2C9	**2*	*rs1799853*
CYP2C9	**3*	*rs1057910*
CYP2C9	**4*	*rs56165452*
CYP2C9	**5*	*rs28371686*
CYP2C9	**6*	*rs9332131*
CYP2C9	**8/*27*	*rs7900194*
CYP2C9	**11*	*rs28371685*
CYP2C9	**12*	*rs9332239*
CYP2C9	**13*	*rs72558187*
CYP2C9	**15*	*rs72558190*
CYP2C9	**25*	*rs869277704*
CYP2D6	**2*	*rs1135840*
CYP2D6	**2/*17/…*	*rs16947*
CYP2D6	**2/*41*	*rs28371725*
CYP2D6	**3*	*rs35742686*
CYP2D6	**4*	*rs3892097*
CYP2D6	**4/*10/…*	*rs1065852*
CYP2D6	**5*	*CYP2D6del*
CYP2D6	**6*	*rs5030655*
CYP2D6	**7*	*rs5030867*
CYP2D6	**8/*14*	*rs5030865*
CYP2D6	**9*	*rs5030656*
CYP2D6	**11*	*rs201377835*
CYP2D6	**12*	*rs5030862*
CYP2D6	**15*	*rs774671100*
CYP2D6	**17*	*rs28371706*
CYP2D6	**18*	*Dup4125_4133*
CYP2D6	**19*	*rs72549353*
CYP2D6	**20*	*rs72549354*
CYP2D6	**29*	*rs59421388*
CYP2D6	**36*	*rs28371735*
CYP3A4	**2*	*rs55785340*
CYP3A4	**17*	*rs4987161*
CYP3A4	**22*	*rs35599367*
CYP3A5	**2*	*rs28365083*
CYP3A5	**3*	*rs776746*
CYP3A5	**7*	*rs41303343*
CYP4F2	**3*	*rs2108622*
DPYD	**2*	*rs3918290*
DPYD	**13*	*rs55886062*
DPYD	*rs67376798 A*	*rs67376798*
OPRM1	*A118G*	*rs1799971*
POR	**28*	*rs1057868*
SLCO1B1	**5*	*rs4149056*
TPMT	**2*	*rs1800462*

TPMT	**3A/*3C*	*rs1142345*
TPMT	**3A/*3B*	*rs1800460*
TPMT	**4*	*rs1800584*
VKORC1	*-1639 A*	*rs9923231*

Document S1: Sample report from SONOGEN XP in three different versions (“comprehensive”, “brief” and “explanation”)

see pdf file attached at the end of this document

Table S2: CYP2C19 inhibitors according to mediQ.ch

**Active Substance**	**Inhibition** 2=moderately strong, 3=strong
armodafinil	2
cannabidiole	2
chloramphenicole	2
clinafloxacine	2
dasabuvir	2
desmethoxyyangonine	2
eslicarbazepine	2
eslicarbazepinacetate	2
esomeprazole	2
ethinylestradiol	2
felbamate	2
fish oil	2
fluconazole	3
fluoxetine	3
fluvoxamine	3
isoniazide	2
kava	2
maribavir	2
meropeneme	2
mestranol	2
moclobemide	2
modafinil	2
omeprazole	2
oxcarbazepine	2
piperaquine	2
stiripentole	2
sultiam	2
topiramate	2

Table S3: CYP2D6 inhibitors according to mediQ.ch

Active Substance	Inhibition<br>2=moderately strong, 3=strong
abirateron	3
asunaprevir	2
budipin	2
cannabidiole	2
chlorphenamine	2
clobazame	2
dapoxetine	2
darunavir-ritonavir	2
dimenhydrinate	2
lorcaserin	2
amodiaquine	2
maribavir	2
mirabegrone	2
peginterferon alf a-2b	2
resveratrole	2
ajmaline	2
amiodarone	2
bupranolole	2
bupropione	3
celecoxib	2
chinidine	3
chloroquine	2
chlorpromazine	2
cimetidine	2
cinacalcet	2
citaloprame	2
clomipramine	2
cocaine	2
darifenacine	2
deramciclane	2
diphenhydramine	2
duloxetine	2
escitaloprame	2
flecainide	2
fluoxetine	3
gefitinib	2
halofantrine	2
haloperidole	2
hydroxychloroquine	2
kava	2
levomepromazine	2
ecstasy	2
melperone	2
metoclopramide	2
midodrine	2
moclobemide	2
norfluoxetine	2
orphenadrine	3
paroxetine	3
perazine	2
promethazine	2
propafenone	2
propoxyphene	2
ritonavir	2
terbinafine (systemic)	2
thiodirazine	3
timolole (systemic)	2

trifluperidole	2
saquinavir-ritonavir	2
tizanidine	3
cinnamon	2

Table S4: Drug-gene pairs and relevance class according to PharmGKB

Drug	Therapeutic area	Gene	Relevance class
pimozide	psychiatry	*CYP2D6*	required
tetrabenazine	neurology	*CYP2D6*	required
siponimod	neurology	*CYP2C9*	required
atazanavir	infectiology	*UGT1A1, (CYP2C19)*	recommended
azathioprine	rheumatology	*TPMT1, NUDT15*	recommended
mercaptopurine	oncology	*TPMT1, NUDT15*	recommended
amitriptyline	psychiatry	*CYP2D6, CYP2C19*	actionable
aripiprazole	psychiatry	*CYP2D6*	actionable
atomoxetine	psychiatry	*CYP2D6*	actionable
atorvastatine	cardiology	*SLCO1B1*	actionable
brexpiprazole	psychiatry	*CYP2D6*	actionable
capecitabine	oncology	*DPYD*	actionable
carisoprodole	rheumatology	*CYP2C19*	actionable
carvedilole	cardiology	*CYP2D6*	actionable
celecoxib	rheumatology	*CYP2C9*	actionable
cevimeline	autoimmune disease	*CYP2D6*	actionable
citaloprame	psychiatry	*CYP2C19, (CYP2D6)*	actionable
clobazame	neurology	*CYP2C19*	actionable
clomipramine	psychiatry	*CYP2D6, CYP2C19*	actionable
clopidogrel	cardiology	*CYP2C19*	actionable
clozapine	psychiatry	*CYP2D6*	actionable
codeine	pain therapy	*CYP2D6, OPRM1, CYP3A4*	actionable
darifenacine	urology	*CYP2D6*	actionable
desipramine	psychiatry	*CYP2D6, CYP2C19*	actionable
doxepine	psychiatry	*CYP2D6, (CYP2C19)*	actionable
efavirenz	infectiology	*CYP2B6*	actionable
fesoterodine	urology	*CYP2D6*	actionable
fluorouracil	oncology	*DPYD*	actionable
iloperidone	psychiatry	*CYP2D6*	actionable
imipramine	psychiatry	*CYP2C6, CYP2C19*	actionable
nortriptyline	psychiatry	*CYP2D6, CYP2C19*	actionable
pantoprazole	gastroenterology	*CYP2C19*	actionable
perphenazine	psychiatry	*CYP2D6*	actionable
phenytoine	neurology	*CYP2C9*	actionable
propafenone	cardiology	*CYP2D6*	actionable
simvastatin	cardiology	*SLCO1B1*	actionable
tamoxifen	oncology	*CYP2D6, CYP3A4*	actionable
thioridazine	psychiatry	*CYP2D6*	actionable
tioguanine	oncology	*TPMT1, NUDT15*	actionable
tramadole	pain therapy	*CYP2D6*	actionable
trimipramine	psychiatry	*CYP2D6, CYP2C19*	actionable
voriconazole	infectiology	*CYP2C19*	actionable
vortioxetine	psychiatry	*CYP2D6*	actionable
warfarin	cardiology	*CYP2C9, VKORC1, CYP4F2*	actionable
acenoumarole	cardiology	*CYP2C9, VKORC1,CYP4F2*	informative
diclofenac	rheumatology	*CYP2C9*	informative
escitaloprame	psychiatry	*CYP2C19*	informative
flecainide	cardiology	*CYP2D6*	informative
fluribiprofene	rheumatology	*CYP2C9*	informative
fluvoxamine	psychiatry	*CYP2D6*	informative
haloperidole	psychiatry	*CYP2D6*	informative
ibuprofen	rheumatology	*CYP2C9*	informative
lansoprazole	gastroenterology	*CYP2C19*	informative
methoxyflurane	anaesthesiology	*CACNA1S, RYR1*	informative
metoprolol	cardiology	*CYP2D6*	informative

mirtazapine	psychiatry	*CYP2D6 (CYP1A2, CYP3A4)*	informative
morphine	pain therapy	*OPRM1*	informative
olanzapine	psychiatry	*CYP1A2, (CYP2D6)*	informative
omeprazole	gastroenterology	*CYP2C19*	informative
ondansetrone	oncology	*CYP2D6*	informative
oxycodone	pain therapy	*CYP2D6 (CYP2C19)*	informative
paroxetine	psychiatry	*CYP2D6*	informative
phenprocoumon	cardiology	*CYP2C9, VKORC1,CYP4F2*	informative
piroxicam	rheumatology	*CYP2C9*	informative
propofole	anaesthesiology	*CYP2B6*	informative
risperidone	psychiatry	*CYP2D6*	informative
rosuvastatin	cardiology	*SLCO1B1*	informative
sertraline	psychiatry	*CYP2C19*	informative
tacrolismus	transplantation	*CYP3A5, POR (CYP3A4)*	informative
tropisetrone	oncology	*CYP2D6*	informative
venlafaxine	psychiatry	*CYP2D6*	informative
zuclopenthixole	psychiatry	*CYP2D6*	informative

Table S5: Additional recommended changes for current co-medication

Drug	Phenotype variant	n patients	Drugs triggering PGx testing	Clinical recommendation to change triggering drugs
metoprolol	*CYP2D6* IM	4	clopidogrel	1
metoprolol	*CYP2D6* IM	1	opioids	1
metoprolol	*CYP2D6* UM	1	clopidogrel	0
atorvastatin	*SLCO1B1* decreased function	1	opioids	1
atorvastatin	*SLCO1B1* decreased function	3	clopidogrel	1
rosuvastatin	*SLCO1B1* decreased function	1	clopidogrel	0
simvastatin	*SLCO1B1* decreased function	1	clopidogrel	1
phenprocoumon	*CYP2C9* NM, *CYP4F2* PM, *VKORC1* decreased function	1	clopidogrel	1
phenprocoumon	*CYP2C9* NM, *CYP4F2* PM, *VKORC1* normal function	1	clopidogrel	1
phenprocoumon	*CYP2C9* PM, *CYP4F2* NM, *VKORC1* decreased function	1	clopidogrel	1
phenprocoumon	*CYP2C9* IM, *CYP4F2* IM, *VKORC1* decreased function	2	clopidogrel	1
phenprocoumon	*CYP2C9* NM, *CYP4F2* NM, *VKORC1* decreased function	1	clopidogrel	1
oxycodone	*CYP2D6* IM	2	clopidogrel	0
oxycodone	*CYP2D6* IM	1	opioids	1
oxycodone	*CYP2D6* IM	1	polypsychopharmacotherapy	0
tramadol	*CYP2D6* IM	1	polypsychopharmacotherapy	0
pantoprazole	*CYP2C19* UM	1	opioids	1
pantoprazole	*CYP2C19* UM	2	clopidogrel	0
tacrolimus	*CYP3A5* NM	1	opioids	0
flupenthixole	*CYP2D6* IM	1	polypsychopharmacotherapy	1
sertraline	*CYP2C19* UM	1	polypsychopharmacotherapy	0
bisoprolol	*CYP2D6* IM	1	screening	na, PGx screening
atorvastatin	*CYP2D6* IM	1	screening	na, PGx screening
amitriptyline	*CYP2D6* PM	1	screening	na, PGx screening

SONOGEN XP report for Annemarie-Clara Muster - comprehensive version

**First name:**	Annemarie-Clara	**Laboratory sample ID:**	12345
**Last name:**	Muster	**Sample collection date:**	October 22, 2020
**Date of birth:**	April 17, 1975	**Report date:**	March 1, 2021
**Gender:**	female		
**Treatment:**	clopidogrel, ibuprofen, pantoprazole, pregabalin, tamoxifen, tramadol		

1 Report summary

1.1 PGx profile - clinically relevant variants

Gene	Genotype	Predicted phenotype	Effect
CYP2C9	*3/*3	PM*3	very slow metabolism
CYP2C19	*1/*3	IM	slow metabolism
CYP2D6	*4J/*10	IM	slow metabolism
DPYD	*1/HapB3	IM+	slow metabolism
POR	*28/*28	increased function	fast metabolism
VKORC1	-1639GA	decreased function	increased drug efficacy

1.2 Drug - PGx interactions

CR	Active ingredient	Suggested action
E	tamoxifen	CYP2D6 IM:<br>• **Consider alternative hormonal therapy** such as aromatase inhibitor for postmenopausal women or aromatase inhibitor along with ovarian function suppression in premenopausal women.<br>• **If aromatase inhibitor use is contraindicated, consider use of a higher tamoxifen dose (40 mg /day).**<br>• **Avoid concomitant use of CYP2D6 inhibitors** (strong to weak)**.**
D	clopidogrel	CYP2C19 IM:<br>• **Choose alternative antiplatelet therapy** if no contraindication (e.g., prasugrel, ticagrelor).
C	ibuprofen	CYP2C9 PM*3:<br>• **Initiate with 25-50% of lowest starting dose and titrate dose upward to clinical effect or 25-50% of maximum dose.**<br>• **Carefully monitor adverse events or**<br>• **Consider an alternate therapy not metabolized by CYP2C9 or not significantly impacted by CYP2C9 genetic variants.**
B	pantoprazole	CYP2C19 IM:<br>• Initiate standard starting daily dose.<br>• For chronic therapy (>12 weeks) and once efficacy achieved, **consider 50% reduction in daily dose**<br>• **Monitor for continued efficacy.**
B	tramadol	CYP2D6 IM:<br>• **Be alert to decreased efficacy** (symptoms of insufficient pain relief).<br>• **Consider dose increase.**<br>• **If response is still inadequate, select alternative drug**- not oxycodone or codeine-
?	pregabalin	Current literature (e.g. dosing guidelines, drug labels, peer reviewed articles) does not allow PGx-based recommendation.

© 2014 - 2021 INTLAB AG

1.3 Predictable drug - PGx interactions

Only active ingredient - phenotype constellations are considered for which the suggested action has a clinical relevance and the patient’s phenotype is altered.

Gene	Phenotype	Active ingredients
CYP2C9	PM*3	**Antidiabetic drugs:** glyburide<br>**Antiepileptics:** phenytoin<br>**Antiinflammatory agents:** diclofenac, flurbiprofen, piroxicam<br>**Dermatological preparations, other:** diclofenac<br>**Immunosuppressants:** siponimod<br>**NSAIDs:** celecoxib, diclofenac, flurbiprofen, ibuprofen, lornoxicam, meloxicam, piroxicam, tenoxicam<br>**NSAIDs, topical:** diclofenac, flurbiprofen, ibuprofen, piroxicam<br>**Other antineoplastic agents:** celecoxib<br>**Other cardiac preparations:** ibuprofen<br>**Other gynecologicals:** ibuprofen<br>**Throat preparations:** flurbiprofen
CYP2C9<br>CYP4F2<br>VKORC1	PM*3<br>NM<br>decreased function	**Antithrombotic agents:** acenocoumarol, phenprocoumon, warfarin
CYP2C19	IM	**Antidepressants:** citalopram, escitalopram<br>**Antimycotics:** voriconazole<br>**Antithrombotic agents:** clopidogrel<br>**Anxiolytics:** clobazam<br>**Muscle relaxants, centrally acting agents:** carisoprodol<br>**PPIs:** lansoprazole, omeprazole, pantoprazole, tak-390mr
CYP2C19<br>CYP2D6	IM<br>IM	**Antidepressants:** amitriptyline, clomipramine, doxepin, imipramine, trimipramine
CYP2D6	IM	**Antiarrhythmics:** flecainide, propafenone<br>**Antidepressants:** desipramine, nortriptyline, venlafaxine<br>**Antipsychotics:** aripiprazole, perphenazine, pimozide, risperidone, thioridazine, zuclopenthixol<br>**Beta blockers:** metoprolol<br>**Cough suppressants:** codeine<br>**Hormone antagonists and related agents:** tamoxifen<br>**Nervous system drugs, other:** tetrabenazine<br>**Opioids:** oxycodone, tramadol<br>**Parasympathomimetics:** cevimeline<br>**Psychostimulants, agents used for adhd and nootropics:** atomoxetine<br>**Others:** iloperidone
DPYD	IM+	**Antimetabolites:** capecitabine, fluorouracil
POR	increased function	No predictable drug - PGx interaction found

© 2014 - 2021 INTLAB AG

2 Detailed report

2.1 Treatment interactions

clopidogrel

Drug - PGx interactions

clopidogrel with phenotype CYP2C19 IM (CPIC)

**Contextual information**	
SONOGEN	Clopidogrel is a prodrug and the formation of its active metabolites is mainly metabolized by CYP2C19 with contributions of CYP1A2, CYP2B6 and CYP3As. [1][2]<br>Genetic polymorphisms of *CYP2C19* are associated with altered clopidogrel metabolism in healthy volunteers and in patients. [3][4][5][6][7][8][9][10][11]<br><br>The *CYP2C19*2* was identified as a major determinant of prognosis in young patients (aged <45 years) who received clopidogrel treatment after myocardial infarction. [8] Another study showed that carriers of a reduced-function CYP2C19 allele had significantly lower levels of clopidogrel's active metabolite, diminished platelet inhibition, and a higher rate of major adverse cardiovascular events. [9]<br><br>The CYP2C19 IM phenotype leads to reduced metabolism of clopidogrel: reduced platelet inhibition; increased residual platelet aggregation; increased risk for adverse cardiovascular events. [12][13]<br><br>The clinical efficacy of clopidogrel in CYP2C19 IM and PM is nil or at least extremely limited in patients with acute stroke or those with acute coronary syndromes treated with stenting. [14]
**Management**	
D<br>CPIC<br>A	**CPIC dosing guideline**<br>Alternative antiplatelet therapy if no contraindication (e.g., prasugrel, ticagrelor). [12]
D<br>DPWG<br>A	**DPWG dosing guideline**<br>Percutaneous coronary intervention, stroke or TIA: choose an alternative or double the dose to 150 mg/day (600 mg loading dose) Prasugrel, ticagrelor and acetylsalicylic acid/dipyridamole are not metabolised by CYP2C19 (or to a lesser extent).<br><br>Other indication: no action required. [15]

ibuprofen

Drug - PGx interactions

ibuprofen with phenotype CYP2C9 PM*3 (SONOGEN)

**Contextual information**	
SONOGEN	Ibuprofen is administered as a racemic mixture of R (-) and S (+) enantiomers, with S-ibuprofen being largely responsible for its pharmacologic activity. CYP2C9 is the major enzyme involved in the hydroxylation of S (+) ibuprofen, whereas R (-) ibuprofen hydroxylation is catalyzed by CYP2C8 and CYP2C9. [16][17][18]<br><br>The *CYP2C9*3* allele is associated with decreased clearance, increased plasma concentration and prolonged half-life of ibuprofen. [19][20][21][22][16] Several studies showed, that the oral clearance of ibuprofen was reduced by about 45% in *CYP2C9*3/*3* compared to *CYP2C9*1/*1*. [23][21][20][19]<br><br>Because most NSAID adverse events are dose dependent, it is reasonable to assume that elevated exposure increases the risk of adverse events. [24] Several studies found an increased risk of gastric bleeding episodes with NSAIDs, such as ibuprofen, in carriers of variant CYP2C9 alleles (n=218 [25], n=26 [26], n=103 [27], n=188 [28]).<br><br>CYP2C9 PM*3 have a strongly reduced enzyme function and a significantly reduced metabolism and prolonged half-life of ibuprofen. The higher plasma concentrations may increase the probability and/or severity of toxicities. [24]

 © 2014 - 2021 INTLAB AG

Management	
CPIC	**CPIC dosing guideline**<br>Initiate therapy with 25-50% of the lowest recommended starting dose. Titrate dose upward to clinical effect or 25-50% of the maximum recommended dose with caution.<br>In accordance with the prescribing information, use the lowest effective dosage for shortest duration consistent with individual patient treatment goals. Upward titration should not occur until after steady state is reached (at least 5 days). Carefully monitor adverse events such as changes in blood pressure and kidney function during course of therapy.<br><br>Alternatively, consider an alternate therapy not metabolized by CYP2C9 or not significantly impacted by *CYP2C9* genetic variants (such as aspirin, ketorolac, naproxen and sulindac). Selection of therapy will depend on individual patient treatment goals and risks for toxicity. [24]

pantoprazole

Drug - PGx interactions

pantoprazole with phenotype CYP2C19 IM (CPIC)

Contextual information	
SONOGEN	Pantoprazole is metabolized mainly by CYP2C19 and to minor extents by CYP3A4, CYP2D6, and CYP2C9. [29] [1]<br><br>The influence of *CYP2C19* genotypic differences on the pharmacokinetics and pharmacodynamics of proton pump inhibitors (PPIs) is well established. [30] [31] [32] [33] [34] A study showed that 57% of the intersubject variability in pantoprazole clearance can be explained by the *CYP2C19* genotype status. Pantoprazole concentration differed significantly and were the highest in patients with reduced CYP2C19 activity (IM and PM). [35]<br><br>CYP2C19 IMs have decreased enzyme activity. [33] Individuals with slow clearance and higher plasma concentrations of PPIs do experience stronger suppression of gastric acid secretion and improved therapeutic effectiveness and may consider a dose reduction to minimize the risk of toxicity that is associated with long-term PPI use (over exposure). [36] [37] [38] [39]
**Management**	
CPIC	**CPIC dosing guideline**<br>Initiate standard starting daily dose. For chronic therapy (>12 weeks) and once efficacy achieved, consider 50% reduction in daily dose and monitor for continued efficacy. [38] [39]

pregabalin

Drug - PGx interactions

?	Current literature (e.g. dosing guidelines, drug labels, peer reviewed articles) does not allow PGx-based recommendation.

tamoxifen

Drug - PGx interactions

tamoxifen with phenotype CYP2D6 IM (SONOGEN)

© 2014 - 2021 INTLAB AG

Contextual information	
SONOGEN	Tamoxifen is a prodrug that undergoes hepatic metabolism by CYP2D6 and, to a lesser extent, by CYP3A4 to form active metabolites 4-hydroxytamoxifen and endoxifen, which show a 100-fold greater affinity for estrogen receptors than the parent compound. [40]<br><br>*CYP2D6* genotype accounts for a large proportion (30 %) of the variability in endoxifen concentration. [41] Plasma endoxifen concentrations after 4 months of tamoxifen therapy (n=80) were statistically significantly lower in CYP2D6 IMs (43.1 nM, 95% CI = 33.3 to 52.9 nM) than in NMs (78.0 nM, 95%CI = 65.9 to 90.1 nM) (P=0.003). [42]<br><br>The effect of CYP2D6 activity on endoxifen concentration has been well established with many studies demonstrating that reduced enzyme activity results in decreased endoxifen formation. [43] [42] [44] [45] [46] Additionally, CYP2D6 activity and low levels of endoxifen have been associated with higher rates of recurrence or reduced efficacy. [47] [48] [49] [40] [50] [51] [52]<br><br>Other studies (BIG1–98 [53] , ATAC [54]) could not show a link between *CYP2D6* genotype and clinical outcomes with tamoxifen, but these studies provoked criticism due to concerns regarding genotyping error (use of formalin-fixed paraffin-embedded tumor tissue) and the analysis of small subsets of the main trials. [55] [56]<br><br>A study suggests there is an endoxifen threshold concentration that needs to be reached in order to have good clinical outcomes and patients with an impaired CYP2D6 metabolizer phenotype were overrepresented in the group that failed to reach that threshold. [47]<br><br>Dose escalation studies showed that increasing the tamoxifen dose from 20 to 40 mg/day in patients with low-activity CYP2D6 phenotypes (PM or IM) increases endoxifen concentrations without any obvious increases in treatment-related toxicity. After the dose increase for IM, there was no longer a difference in endoxifen concentrations between NM and IM patients. [57] [58] [59]<br><br>CYP2D6 IM have reduced enzyme activity resulting in lower endoxifen concentrations compared to NMs. They have a higher risk of breast cancer recurrence and worse event-free and recurrence-free survival compared to NMs. [60] [51]
**Management**	
E<br>CPIC<br>A	**CPIC dosing guideline**<br>Consider hormonal therapy such as an aromatase inhibitor for postmenopausal women or aromatase inhibitor along with ovarian function suppression in premenopausal women, given that these approaches are superior to tamoxifen regardless of CYP2D6 genotype. [61] If aromatase inhibitor use is contraindicated, consideration should be given to use a higher but FDA approved tamoxifen dose (40 mg/day). [58] Avoid CYP2D6 strong to weak inhibitors. [60]
E<br>DPWG<br>A	**DPWG dosing guideline**<br>Increased risk for relapse of breast cancer. Avoid concomitant use of CYP2D6 inhibitors. Consider aromatase inhibitor for postmenopausal women. [62] [63]

tramadol

Drug - PGx interactions

tramadol with phenotype CYP2D6 IM (SONOGEN)

Contextual information	
SONOGEN	Tramadol is a major substrate of CYP2D6, CYP3A4 and CYP2B6. [1] It is metabolized by CYP2D6 to the pharmacologically active *O*-desmethyltramadol (M1), its main analgesic effective metabolite. [64] The *CYP2D6* genotype is shown to be linked to the concentration of M1, resulting in the different efficacy of tramadol treatment. [65] [66] [67] [68]<br><br>It was shown, that tramadol had lower clearance and longer half life in CYP2D6 IMs compared to NMs. [69]<br><br>CYP2D6 IMs have a decreased enzyme activity compared to NMs and might be at risk for decreased efficacy with standard doses.
**Management**	
B<br>DPWG<br>A	**DPWG Dosing Guideline**<br>It is not possible to provide a recommendation for dose adjustment, because the total analgesic effect changes when the ratio between the mother compound and the active metabolite changes.<br>Be alert to a reduced effectiveness. In the case of inadequate effectiveness try a dose increase or if this does not work choose an alternative. Do not select codeine, as this is also metabolised by CYP2D6. Morphine is not metabolised by CYP2D6. Oxycodone is metabolised by CYP2D6 to a limited extent, but this does not result in differences in analgesia in patients.<br>If no alternative is selected, advise the patient to report inadequate analgesia. [15]

 © 2014 - 2021 INTLAB AG

2.2 PGx profile - complete

Gene	Genotype	Predicted phenotype	PGx organizations	Effect	Tested alleles
CYP2C9	*3/*3	PM*3	SONOGEN	very slow metabolism	*1, *2, *3, *4, *5, *6, *8, *11, *12, *13, *15, *25, *27
CYP2C19	*1/*3	IM	CPIC, DPWG	slow metabolism	*1, *2, *3, *4A, *4B, *5, *6, *7, *8, *17
CYP2D6	*4J/*10	IM	SONOGEN	slow metabolism	*1, *2, *3, *4, *4J, *4K, *4M, *5, *6, *6C, *7, *8, *9, *10, *11, *12, *14A, *14B, *15, *18, *19, *20, *29, *34, *39, *41, *69, CNV
DPYD	*1/HapB3	IM+	SONOGEN	slow metabolism	*1, *2A, *13, 2846T, HapB3
POR	*28/*28	increased function	SONOGEN	fast metabolism	*1, *28
VKORC1	-1639GA	decreased function	SONOGEN	increased drug efficacy	-1639A, -1639G
ABCB1	1236CC, 2677GG, 3435CC	CGC/CGC	SONOGEN	normal drug efficacy	CAC, CAT, CGC, CGT, CTC, CTT, TAC, TAT, TGC, TGT, TTC, TTT
COMT	High /Intermediate	APS	SONOGEN	normal metabolism	High, Intermediate, Low
CYP1A2	*1A/*1A	NM	SONOGEN	normal metabolism	*1A, *1C, *1F, *1K, *1L, *7, *11
CYP2B6	*1/*1	NM	SONOGEN	normal metabolism	*1, *6, *18
CYP3A4	*1/*1	*22 non-carrier	SONOGEN	normal metabolism	*1, *2, *17, *22
CYP3A5	*3/*3	non-expresser	SONOGEN	normal metabolism	*1, *2, *3, *3+2, *6, *7
CYP4F2	*1/*1	NM	SONOGEN	normal metabolism	*1, *3
OPRM1	118AA	normal function	SONOGEN	normal drug efficacy	118A, 118G
SLCO1B1	*1a/*1a	normal function	CPIC	normal drug efficacy	*1a, *5
TPMT	*1/*1	NM	CPIC	normal metabolism	*1, *2, *3A, *3B, *3C, *4

3 Annex

3.1 Disclaimer

The present individual treatment optimization proposal and the related information was generated by SONOGEN XP - a clinical decision support and pharmacogenetic expert system. This software is an in vitro medical device and has been developed according to the directive on in vitro diagnostic medical devices (Directive 98/79/EC of the European Parliament and of the Council). The containing information has been collected and reviewed to our best knowledge, however there is no guarantee that it contains the latest scientific findings and that all adverse or important outcomes will be reported in the literature and integrated in the SONOGEN XP software. The responsibility for a correct drug-treatment prescription lies with the treating physician and the user should always apply his independent professional judgement.

3.2 Limitation

This pharmacogenetic test will not detect all the known mutations of a gene. Absence of a detectable gene mutation does not rule out the possibility of an altered phenotype due to the presence of an undetected mutation or due to other factors influencing the drug efficacy, such as drug-drug-interactions, comorbidities or lifestyle habits.

© 2014 - 2021 INTLAB AG

3.3 Related (major) substrates

Only substrates for gene products are considered for which the patient's phenotype is altered. For CYPs, only major substrates are considered.

Gene	Phenotype	Active ingredients
CYP2C9	PM*3	**Alimentary tract and metabolism:** calcitriol, dronabinol, glimepiride, glipizide, glyburide, lansoprazole, nateglinide, pioglitazone, tolbutamide<br>**Antiinfectives for systemic use:** etravirine, sulfadiazine, sulfisoxazole, trimethoprim, zidovudine<br>**Antineoplastic and immunomodulating agents:** bortezomib, celecoxib, ifosfamide, paclitaxel, siponimod, tamoxifen<br>**Blood and blood forming organs:** acenocoumarol, phenprocoumon, warfarin<br>**Cardiovascular system:** atorvastatin, bosentan, candesartan, diltiazem, fluvastatin, ibuprofen, indomethacin, irbesartan, losartan, nicardipine, olmesartan, torasemide, valsartan, verapamil<br>**Dermatologicals:** calcitriol, diclofenac, ethanol, isotretinoin, terbinafine<br>**Genito urinary system and sex hormones:** estradiol, ibuprofen, naproxen<br>**Musculo-skeletal system:** celecoxib, diclofenac, etodolac, etoricoxib, flurbiprofen, ibuprofen, indomethacin, lornoxicam, mefenamic acid, meloxicam, naproxen, piroxicam, tenoxicam<br>**Nervous system:** cocaine, droperidol, eletriptan, fluoxetine, johanniskraut[cs], perphenazine, phenytoin, propofol, temazepam, valproic acid, zopiclone<br>**Respiratory system:** cocaine, flurbiprofen, montelukast, zafirlukast<br>**Sensory organs:** cocaine, diclofenac, flurbiprofen, indomethacin, piroxicam, sulfisoxazole<br>**Various:** ethanol, tolbutamide<br>**Others:** fosphenytoin, methyltestosterone, oxymorphone, st. john's wort, zileuton
CYP2C19	IM	**Alimentary tract and metabolism:** esomeprazole, gliclazide, lansoprazole, omeprazole, pantoprazole, rabeprazole, tak-390mr<br>**Antiinfectives for systemic use:** dapsone, etravirine, nelfinavir, nevirapine, sulfadiazine, tipranavir, voriconazole<br>**Antineoplastic and immunomodulating agents:** bortezomib, ifosfamide, nilutamide, tacrolimus, tamoxifen, thalidomide, vorinostat<br>**Antiparasitic products, insecticides and repellents:** atovaquone, malathion, pentamidine, proguanil<br>**Blood and blood forming organs:** clopidogrel, ticlopidine<br>**Cardiovascular system:** doxazosin, verapamil<br>**Dermatologicals:** dapsone, ethanol, tacrolimus<br>**Genito urinary system and sex hormones:** estradiol, levonorgestrel, progesterone<br>**Musculo-skeletal system:** carisoprodol<br>**Nervous system:** amitriptyline, citalopram, clobazam, clomipramine, diazepam, doxepin, droperidol, escitalopram, fluoxetine, imipramine, methsuximide, methylphenobarbital, moclobemide, perphenazine, pethidine, phenobarbital, phenytoin, selegiline, sertraline, temazepam, trimipramine<br>**Respiratory system:** oxymetazoline, roflumilast<br>**Various:** ethanol<br>**Others:** fosphenytoin, methyltestosterone
CYP2D6	IM	**Alimentary tract and metabolism:** dolasetron, gliclazide, loperamide, ondansetron, palonosetron, pioglitazone, rabeprazole, ranitidine, tropisetron[cs]<br>**Antiinfectives for systemic use:** ritonavir<br>**Antineoplastic and immunomodulating agents:** bortezomib, doxorubicin, gefitinib, idarubicin, ifosfamide, lomustine, tamoxifen<br>**Antiparasitic products, insecticides and repellents:** malathion, primaquine<br>**Cardiovascular system:** betaxolol, captopril, carteolol, carvedilol, diltiazem, flecainide, indomethacin, lidocaine, metoprolol, mexiletine, nebivolol, nicardipine, pindolol, procainamide, propafenone, propranolol, timolol<br>**Dermatologicals:** diphenhydramine, ethanol, isotretinoin, lidocaine<br>**Genito urinary system and sex hormones:** clomifene, fesoterodine, lisuride, tamsulosin, tolterodine, yohimbine<br>**Musculo-skeletal system:** indomethacin<br>**Nervous system:** almotriptan, amitriptyline, amoxapine, aripiprazole, atomoxetine, benzatropine, buprenorphine, cevimeline, chlorpromazine, clomipramine, clozapine, cocaine, desipramine, dihydrocodeine, donepezil, doxepin, droperidol, duloxetine, flunarizine, fluoxetine, fluphenazine, fluvoxamine, galantamine, haloperidol, imipramine, ketamine, lidocaine, lisuride, loxapine, maprotiline, methamphetamine, mianserin, mirtazapine, moclobemide, nortriptyline, olanzapine, opipramol[cs], oxycodone, paroxetine, pipotiazine, protriptyline, risperidone, tetrabenazine, thioridazine, tramadol, trimipramine, venlafaxine, vortioxetine, zuclopenthixol<br>**Respiratory system:** astemizole, azelastine, cocaine, codeine, dextromethorphan, diphenhydramine, epinastine, hydrocodone, loratadine, promethazine, theophylline<br>**Sensory organs:** azelastine, betaxolol, carteolol, cocaine, epinastine, indomethacin, lidocaine, timolol<br>**Systemic hormonal preparations, excl. sex hormones and insulins:** cinacalcet<br>**Various:** ethanol<br>**Others:** amphetamine, iloperidone, lopinavir, nefazodone, oxymorphone, tiotropium, toremifene
DPYD	IM+	**Antineoplastic and immunomodulating agents:** capecitabine, fluorouracil
POR	increased function	**Dermatologicals:** finasteride<br>**Genito urinary system and sex hormones:** finasteride

3.4 Related inducers

Gene	Phenotype	Active ingredients
ABCB1	CGC/CGC	budesonide, cholecalciferol, cisplatin, cyclosporine, dexamethasone, efavirenz, erlotinib, erythromycin, fexofenadine, flucloxacillin, ginkgo biloba, hydroxyurea, nelfinavir, oxaliplatin, oxcarbazepine, paclitaxel, phenobarbital, phenytoin, prednisolone, prednisone, reserpine, rifampicin, ritonavir, sildenafil, st. john's wort, tamoxifen, tipranavir, tolbutamide, topiramate, trazodone, ursodeoxycholic acid, valproic acid, verapamil, vinblastine, vincristine, vitamin a, vitamin e, yohimbine
COMT	APS	No related inducer found
CYP1A2	NM	aminoglutethimide, amiodarone, bortezomib, caffeine, carbamazepine, dexamethasone, ginkgo biloba, griseofulvin, infliximab, insulin regular, johanniskraut[cs], lansoprazole, milnacipran, mirtazapine, modafinil, moricizine, nafcillin, nelfinavir, nicardipine, omeprazole, orphenadrine, oxazepam, pantoprazole, phenobarbital, primaquine, primidone, propofol, rifabutin, rifampicin, ritonavir, secobarbital, st. john's wort, tak-390mr, tipranavir, triamterene, vorinostat
CYP2B6	NM	abacavir, atorvastatin, carbamazepine, clotrimazole, conjugated estrogens, cyclophosphamide, darunavir, dexamethasone, efavirenz, fosamprenavir, fosphenytoin, ginkgo biloba, lovastatin, metamizole, methimazole, milnacipran, modafinil, nelfinavir, nevirapine, nicotine, nilotinib, omeprazole, orphenadrine, phenobarbital, phenytoin, pioglitazone, pravastatin, primidone, rifampicin, ritonavir, roflumilast, rosiglitazone, rosuvastatin, simvastatin, ticagrelor, tipranavir
CYP2C9	PM*3	aprepitant, bosentan, carbamazepine, caspofungin, colchicine, cyclophosphamide, dapsone, dexamethasone, folic acid, fosaprepitant, fosphenytoin, ginseng, griseofulvin, ifosfamide, johanniskraut[cs], milnacipran, nafcillin, nelfinavir, nilotinib, peginterferon alfa-2a, peginterferon alfa-2b, phenobarbital, phenytoin, primidone, propofol, raloxifene, rifampicin, rifapentine, ritonavir, rosuvastatin, secobarbital, sildenafil, st. john's wort, ticagrelor, warfarin
CYP2C19	IM	acetylsalicylic acid, aminoglutethimide, bosentan, carbamazepine, caspofungin, dexamethasone, fosphenytoin, ginkgo biloba, johanniskraut[cs], milnacipran, nelfinavir, norethindrone, phenobarbital, phenytoin, prednisone, rifampicin, ritonavir, st. john's wort
CYP2D6	IM	buprenorphine, carbamazepine, ginkgo biloba, nicotine, phenobarbital, propofol

 © 2014 - 2021 INTLAB AG

CYP3A4	*22 non-carrier	aminoglutethimide, aprepitant, artemether, bexarotene, bezafibrate, bicalutamide, bosentan, budesonide, buprenorphine, buspirone, calcitriol, carbamazepine, caspofungin, cholecalciferol, cisplatin, colchicine, conjugated estrogens, cyclophosphamide, deferasirox, dexamethasone, diclofenac, dicloxacillin, efavirenz, eletriptan, estradiol, etoposide, etoricoxib, etravirine, felbamate, fenofibrate, flucloxacillin, fosamprenavir, fosaprepitant, fosphenytoin, ginkgo biloba, ginseng, glimepiride, griseofulvin, ifosfamide, infliximab, johanniskraut[cs], lamotrigine, medroxyprogesterone acetate, metamizole, metyrapone, midazolam, milnacipran, modafinil, moricizine, nafcillin, nelfinavir, nevirapine, oxcarbazepine, paclitaxel, pantoprazole, pazopanib, pentobarbital, phenobarbital, phenytoin, pioglitazone, prednisolone, prednisone, primidone, propofol, rifabutin, rifampicin, rifapentine, rifaximin, ritonavir, rosiglitazone, rosuvastatin, rufinamide, sildenafil, st. john's wort, tamoxifen, terbinafine, topiramate, ursodeoxycholic acid, valproic acid, vinblastine, vincristine, vitamin e
CYP3A5	non-expresser	budesonide, deferasirox, ginkgo biloba, infliximab, milnacipran, oxcarbazepine
CYP4F2	NM	No related inducer found
DPYD	IM+	No related inducer found
OPRM1	normal function	No related inducer found
POR	increased function	No related inducer found
SLCO1B1	normal function	phenobarbital, rifampicin
TPMT	NM	phenobarbital
VKORC1	decreased function	No related inducer found

3.5 Related inhibitors

Gene	Phenotype	Active ingredients
ABCB1	CGC/CGC	**yes:** abacavir, amiodarone, amitriptyline, amlodipine, amodiaquine, atazanavir, atorvastatin, atovaquone, benzocaine, buprenorphine, carvedilol, cimetidine, cisapride, clarithromycin, cyclosporine, dasatinib, delavirdine, dexamethasone, diltiazem, dronedarone, eribulin, erythromycin, escitalopram, esomeprazole, estradiol, everolimus, felodipine, ginkgo biloba, ginseng, imatinib, itraconazole, ixabepilone, ketoconazole, lansoprazole, lapatinib, levothyroxine, lidocaine, loratadine, lovastatin, mefloquine, methadone, micafungin, mifepristone, nevirapine, nicardipine, nifedipine, nilotinib, nisoldipine, nitrendipine, ofloxacin, olanzapine, omeprazole, oxybutynin, paliperidone, pantoprazole, phenobarbital, phenytoin, pimozide, posaconazole, pravastatin, progesterone, propafenone, propranolol, quetiapine, quinidine, quinine, rabeprazole, ranitidine, ranolazine, reserpine, risperidone, ritonavir, rosiglitazone, saquinavir, sildenafil, sirolimus, solifenacin, sorafenib, st. john's wort, sunitinib, tamoxifen, telaprevir, temsirolimus, ticagrelor, tipranavir, tolterodine, tolvaptan, toremifene, trimipramine, valproic acid, venlafaxine, verapamil, vortioxetine
COMT	APS	**yes:** entacapone, nelfinavir
CYP1A2	NM	**strong:** carbamazepine, fluvoxamine, lidocaine, mexiletine, ofloxacin, primaquine, quinine, sulindac, thiabendazole, zileuton<br>**moderate:** amitriptyline, amlodipine, amphetamine, chloroquine, cimetidine, ciprofloxacin, diclofenac, duloxetine, epinephrine, ethanol, fluoxetine, gemfibrozil, levofloxacin, medroxyprogesterone acetate, methimazole, methoxsalen, miconazole, modafinil, nelfinavir, nifedipine, nisoldipine, norfloxacin, omeprazole, ondansetron, orphenadrine, praziquantel, propofol, protriptyline, ropinirole, tioconazole, tranylcypromine<br>**weak:** albendazole, alosetron, amiodarone, anastrozole, apomorphine, atazanavir, atomoxetine, azacitidine, bortezomib, bromocriptine, buprenorphine, caffeine, citalopram, clarithromycin, clotrimazole, clozapine, conjugated estrogens, delavirdine, dexmedetomidine, disopyramide, disulfiram, entacapone, erythromycin, escitalopram, estradiol, fluconazole, fluphenazine, flutamide, fluvastatin, ginkgo biloba, ginseng, imipramine, interferon alfa-2a, recombinant, interferon alfa-2b, recombinant, interferon gamma-1b, irbesartan, isoniazid, johanniskraut[cs], ketoconazole, lomefloxacin, losartan, memantine, mirtazapine, moclobemide, nefazodone, nevirapine, olanzapine, oxybutynin, pantoprazole, paroxetine, peginterferon alfa-2a, peginterferon alfa-2b, pentoxifylline, perphenazine, propafenone, propranolol, ranitidine, selegiline, sertraline, sildenafil, st. john's wort, tacrine, tenofovir, theophylline, thioridazine, ticagrelor, ticlopidine, tocainide, venlafaxine, verapamil, zafirlukast
CYP2B6	NM	**strong:** cisplatin, ginkgo biloba, memantine, miconazole, orphenadrine, thiotepa, ticlopidine<br>**moderate:** amlodipine, atorvastatin, clopidogrel, desipramine, doxorubicin, duloxetine, methimazole, mifepristone, nitric oxide, oxaliplatin, paroxetine, sertraline, sorafenib, toremifene, zileuton<br>**weak:** amiodarone, azelastine, buprenorphine, citalopram, clotrimazole, disulfiram, fluoxetine, fluvoxamine, isoflurane, ketoconazole, nefazodone, nelfinavir, pazopanib, prasugrel, tamoxifen, venlafaxine
CYP2C9	PM*3	**strong:** amiodarone, candesartan, capecitabine, ciprofloxacin, clarithromycin, delavirdine, econazole, esomeprazole, fluconazole, fluorouracil, flurbiprofen, gemfibrozil, glyburide, ibuprofen, indomethacin, irbesartan, johanniskraut[cs], levofloxacin, losartan, mefenamic acid, miconazole, montelukast, nicardipine, phenprocoumon, piroxicam, sitaxentan, st. john's wort, sulfadiazine, sulfisoxazole, telmisartan, tolbutamide, valproic acid<br>**moderate:** adefovir dipivoxil, amitriptyline, amlodipine, amodiaquine, atazanavir, atorvastatin, azelastine, bortezomib, clomipramine, clozapine, cyclizine, diltiazem, dimethyl sulfoxide, efavirenz, eltrombopag, epinephrine, ethanol, felodipine, fenoprofen, fluvastatin, fluvoxamine, ginkgo biloba, human serum albumin, imipramine, isoniazid, itraconazole, ketoconazole, lansoprazole, malathion, medroxyprogesterone acetate, methimazole, modafinil, nifedipine, nilotinib, nortriptyline, omeprazole, ondansetron, orphenadrine, pantoprazole, piperazine, prasugrel, progesterone, protriptyline, pyrimethamine, quinine, rabeprazole, sorafenib, tenofovir, tenoxicam, ticagrelor, toremifene, trimethoprim, verapamil, voriconazole, warfarin, zafirlukast, zileuton<br>**weak:** anastrozole, aprepitant, atomoxetine, bicalutamide, buprenorphine, caffeine, chloramphenicol, cholecalciferol, cimetidine, cisplatin, clopidogrel, clotrimazole, cyclosporine, dexmedetomidine, dextropropoxyphene, diclofenac, disulfiram, dronedarone, entacapone, eprosartan, escitalopram, estradiol, etodolac, etoposide, etravirine, fenofibrate, fluoxetine, fluphenazine, fosaprepitant, ginseng, imatinib, indinavir, irinotecan, ketoprofen, leflunomide, lovastatin, meloxicam, memantine, methoxsalen, metronidazole, midazolam, nateglinide, nelfinavir, olanzapine, oxaliplatin, oxybutynin, paroxetine, pioglitazone, pravastatin, promethazine, propofol, quinidine, ritonavir, rosiglitazone, saquinavir, selegiline, sertraline, sildenafil, simvastatin, tak-390mr, tamoxifen, teniposide, thioridazine, ticlopidine, tranylcypromine, tretinoin, triazolam, valsartan, zonisamide
CYP2C19	IM	**strong:** atorvastatin, chloramphenicol, clomipramine, clopidogrel, delavirdine, doxepin, erythromycin, esomeprazole, fluconazole, fluorouracil, ginkgo biloba, lansoprazole, lovastatin, miconazole, modafinil, nicardipine, omeprazole, pantoprazole, phenobarbital, proguanil, quinine, rabeprazole, simvastatin, sitaxentan, ticlopidine, zidovudine<br>**moderate:** amitriptyline, azelastine, bortezomib, cimetidine, clozapine, cyclosporine, desipramine, dimethyl sulfoxide, duloxetine, efavirenz, ethanol, felbamate, fluoxetine, fluvastatin, fluvoxamine, gemfibrozil, isoniazid, itraconazole, loratadine, malathion, methimazole, nelfinavir, nortriptyline, orphenadrine, oxcarbazepine, piperazine, prasugrel, progesterone, propofol, protriptyline, sertraline, sirolimus, solifenacin, sorafenib, tacrolimus, tak-390mr, tioconazole, topiramate, toremifene, tranylcypromine, valproic acid, voriconazole, zafirlukast, zileuton<br>**weak:** amiodarone, amprenavir, apixaban, apomorphine, aprepitant, bicalutamide, buprenorphine, cholecalciferol, citalopram, clotrimazole, diazepam, disopyramide, entacapone, escitalopram, estradiol, ethotoin, etravirine, fenofibrate, fosamprenavir, fosaprepitant, gefitinib, ginseng, imipramine, indinavir, indomethacin, johanniskraut[cs], ketoconazole, letrozole, losartan, memantine, methoxsalen, methsuximide, methylphenobarbital, moclobemide, nilutamide, norethindrone, olanzapine, paroxetine, pentamidine, pimozide, pioglitazone, pravastatin, probenecid, ritonavir, rosiglitazone, saquinavir, selegiline, sildenafil, st. john's wort, telmisartan, torasemide, warfarin, zonisamide

CYP2D6	IM	**strong:** azelastine, buprenorphine, bupropion, chlorpromazine, cinacalcet, cisapride, cocaine, delavirdine, dexmedetomidine, flecainide, fluoxetine, fluphenazine, hydroxyzine, johanniskraut[cs], memantine, metoclopramide, miconazole, nicardipine, orphenadrine, oxybutynin, paroxetine, perphenazine, pimozide, piperazine, propafenone, quinidine, ritonavir, st. john's wort, terbinafine, trospium<br>**moderate:** amiodarone, amitriptyline, amlodipine, amodiaquine, amphetamine, atomoxetine, atorvastatin, caffeine, chloroquine, cimetidine, clomipramine, clozapine, darifenacin, desipramine, diltiazem, diphenhydramine, dolasetron, doxepin, dronedarone, duloxetine, epinephrine, escitalopram, esomeprazole, felodipine, fluvastatin, fluvoxamine, ginkgo biloba, haloperidol, hydroxychloroquine, hydroxyurea, idarubicin, imatinib, imipramine, isoniazid, itraconazole, ketoconazole, lansoprazole, lidocaine, lopinavir, loratadine, lumefantrine, methadone, methimazole, methotrimeprazine, mifepristone, nelfinavir, nifedipine, nilotinib, omeprazole, ondansetron, paliperidone, pioglitazone, prasugrel, proguanil, protriptyline, pyrimethamine, quinine, rabeprazole, ranolazine, ropinirole, ropivacaine, sertraline, sirolimus, tacrolimus, thioridazine, thiothixene, ticlopidine, tipranavir, tranylcypromine, trazodone, triprolidine, tropisetron[cs], verapamil, ziprasidone<br>**weak:** acebutolol, asenapine, astemizole, betaxolol, bicalutamide, biperiden, bortezomib, celecoxib, chloramphenicol, chlorphenamine, cholecalciferol, citalopram, clemastine, clotrimazole, codeine, cyclizine, cyclosporine, desloratadine, desvenlafaxine, dextromethorphan, dextropropoxyphene, dimethyl sulfoxide, disulfiram, doxorubicin, entacapone, epinastine, estradiol, fexofenadine, fosamprenavir, gefitinib, ginseng, hydrocodone, indinavir, irbesartan, lomustine, lovastatin, medroxyprogesterone acetate, mefloquine, methoxsalen, methylphenidate, metoprolol, moclobemide, nefazodone, nevirapine, nortriptyline, octreotide, olanzapine, oxprenolol, oxycodone, pantoprazole, pazopanib, pentamidine, pindolol, pravastatin, praziquantel, primaquine, promethazine, propofol, propranolol, ranitidine, reboxetine, risperidone, rosiglitazone, saquinavir, selegiline, sildenafil, simvastatin, tak-390mr, telithromycin, temsirolimus, timolol, valproic acid, venlafaxine, vinblastine, vinorelbine, yohimbine, zafirlukast, zileuton
CYP3A4	*22 non-carrier	**strong:** amprenavir, bicalutamide, boceprevir, buprenorphine, chloramphenicol, delavirdine, diltiazem, disopyramide, doxycycline, erythromycin, everolimus, indinavir, itraconazole, johanniskraut[cs], ketoconazole, lopinavir, methimazole, miconazole, midazolam, mifepristone, nefazodone, nelfinavir, nicardipine, oxybutynin, piperazine, posaconazole, propofol, quinidine, ritonavir, saquinavir, st. john's wort, telaprevir, telithromycin, toremifene, voriconazole, zafirlukast, zolpidem<br>**moderate:** acetaminophen, adefovir dipivoxil, aliskiren, amiodarone, amlodipine, amphetamine, aprepitant, astemizole, atazanavir, atomoxetine, atorvastatin, atovaquone, azelastine, bromocriptine, caffeine, cimetidine, ciprofloxacin, clarithromycin, clindamycin, clotrimazole, conivaptan, cyclosporine, cytarabine, darunavir, dasatinib, desipramine, dexamethasone, dimethyl sulfoxide, dronedarone, duloxetine, efavirenz, epinephrine, ergonovine, ergotamine, eribulin, erlotinib, esomeprazole, felodipine, fentanyl, fluconazole, fluoxetine, fluvastatin, fosamprenavir, fosaprepitant, ginkgo biloba, haloperidol, human serum albumin, hydralazine, ifosfamide, imatinib, imipramine, irinotecan, isoniazid, isradipine, ketamine, lansoprazole, lapatinib, levofloxacin, lidocaine, maraviroc, medroxyprogesterone acetate, mefloquine, micafungin, milnacipran, modafinil, nifedipine, nilotinib, nisoldipine, nitrendipine, nitric oxide, norfloxacin, nortriptyline, octreotide, omeprazole, ondansetron, orphenadrine, paliperidone, pantoprazole, phenelzine, pimozide, pioglitazone, prasugrel, primaquine, propericiazine, protriptyline, rabeprazole, raloxifene, ropinirole, rosiglitazone, sertraline, simvastatin, sitaxentan, tacrolimus, tenofovir, tetracycline, tioconazole, tipranavir, topotecan, tropisetron[cs], valproic acid, verapamil, ziprasidone<br>**weak:** acetazolamide, anastrozole, apomorphine, azithromycin, betamethasone, bortezomib, budesonide, chenodeoxycholic acid, chlorzoxazone, cisapride, clemastine, clozapine, cocaine, cyclophosphamide, danazol, darifenacin, dexmedetomidine, dextropropoxyphene, diazepam, diclofenac, dihydroergotamine, disulfiram, docetaxel, doxorubicin, entacapone, escitalopram, estradiol, ethanol, etoposide, etoricoxib, fluvoxamine, gemfibrozil, ginseng, glyburide, irbesartan, lomustine, losartan, lovastatin, memantine, methadone, methoxsalen, methylprednisolone, metronidazole, mirtazapine, mitoxantrone, nevirapine, olanzapine, oxycodone, paroxetine, pazopanib, pentamidine, pilocarpine, pravastatin, prednisolone, progesterone, quinine, raltegravir, ranolazine, reboxetine, risperidone, selegiline, sildenafil, sirolimus, tak-390mr, tamoxifen, temsirolimus, teniposide, testosterone, ticagrelor, ticlopidine, tranylcypromine, trazodone, trospium, venlafaxine, vinblastine, vincristine, vinorelbine
CYP3A5	non-expresser	**strong:** boceprevir, erythromycin, ritonavir, verapamil<br>**moderate:** amlodipine, cimetidine, clarithromycin, darunavir, dronedarone, duloxetine, erlotinib, fosamprenavir, milnacipran, paliperidone, prasugrel, sitaxentan, tipranavir<br>**weak:** amprenavir, budesonide, indinavir
CYP4F2	NM	**strong:** fenofibrate, ketoconazole<br>**weak:** johanniskraut[cs], st. john's wort
DPYD	IM+	No related inhibitor found
OPRM1	normal function	No related inhibitor found
POR	increased function	No related inhibitor found
SLCO1B1	normal function	**yes:** eltrombopag, pazopanib, rosuvastatin
TPMT	NM	No related inhibitor found
VKORC1	decreased function	**yes:** phenprocoumon, warfarin

3.6 Legend

Biomarker Relevance (BR)

4	Genetic testing required. The drug label states that a genetic testing should be conducted before using this drug. This requirement may only be for a subset of patients. If the drug label states a test "should be" performed, this is to be interpreted as a requirement. [70]
3	Genetic testing recommended. The drug label states that a genetic testing is recommended before using this drug. This recommendation may only be for a subset of patients. If the drug label states a test "should be considered", this is to be interpreted as a recommendation. [70]
2	Actionable PGx. The drug label does not discuss testing for gene variants, but does contain information about changes in efficacy, dosage or toxicity (due to such variants). The drug label may mention contraindication of the drug in a subset of patients but does not require or recommend genetic testing. [70]
1	Informative PGx. The drug label mentions a gene/protein is involved in the metabolism or pharmacodynamics of the drug but gives no information to suggest that variation in this gene/protein leads to a different response. [70]

Clinical Relevance (CR)

F	Clinical effect: **death; arrhythmia; unanticipated myelosuppression**. [71]
E	Clinical effect: **failure of lifesaving therapy**: e.g. anticipated myelosuppression; prevention of cancer relapse; life-threatening complications from diarrhea. [71]
D	Clinical effect: **long-standing discomfort (>168 h), permanent symptom or invalidating injury**: e.g. failure of prophylaxis of atrial fibrillation; venous thromboembolism; decreased inhibition of platelet aggregation; severe diarrhea; hepatic failure; INR > 6.0. [71]

© 2014 - 2021 INTLAB AG

C	Clinical effect: **long-standing discomfort (48-168 h) without permanent injury**: e.g. failure of therapy with antidepressants, atypical antipsychotic drugs; extrapyramidal side effects; bradycardia; parkinsonism; dizziness; somnolence; INR 4.5-6.0. [71]
B	Clinical effect: **short-lived discomfort (< 48 h) without permanent injury**: e.g. reduced decrease in resting heart rate; reduction in exercise tachycardia; decreased pain relief; decreased appetite; insomnia; sleep disturbance; moderate diarrhea not affecting daily activities. [71]
A	Minor clinical effect: e.g. QTc prolongation (< 450 ms female, < 470 ms male); INR increase < 4.5. [71]
✓	Drug-PGx-analysis gives "normal" genotype/phenotype relation.<br>OR genotype/phenotype requires no specific dosing adjustment (i.e. follow drug label dosing recommendation).
?	Missing phenotype information or current literature (e.g. dosing guidelines, drug labels, peer reviewed articles) does not allow PGx-based recommendation.

Level of Evidence (LoE)

E	The variant-drug combination is based on published incomplete case reports, non-significant studies or in vitro, molecular or functional assay evidence only. [71]
D	The variant-drug combination is based on published case reports, well documented, and having relevant pharmacokinetic or clinical endpoints. [71]
C	The variant-drug combination shows moderate evidence of an association (it is replicated but there may be some studies that do not show statistical significance, and/or the effect size may be small). [72] [71]<br>Or drug label information on PGx relevant genes with potential influence on pharmacokinetics, without information on specific variants.
B	The variant-drug combination shows good evidence of an association (it is replicated in more than one cohort with significant p-values, and preferably will have a strong effect size). [72] [71]<br>Or drug label information on specific variants of PGx relevant genes with potential influence on pharmacokinetics.<br>Or the variant-drug combination and recommendation are reflected in peer reviewed articles.
A	The variant-drug combination is reflected in a pharmacogenetic guideline (e.g. CPIC, DPWG), or implemented at a pharmacogenomic research network site (e.g. www.warfarindosing.org) or in another major health system. [72]<br>Or FDA box warning.<br>Or FDA drug label recommendation on pharmacogenetic testing.

PGx - Phenotype

APS	average pain sensitivity
HPS	high pain sensitivity
IM	intermediate metabolizer
IM+	intermediate metabolizer with higher enzyme activity than IM
IM*2	IM with one *2 allele or equivalent (*8, *11, *12)
IM*3	IM with one *3 allele or equivalent (*4, *5, *6, *13, *14, *15, *25)
LPS	low pain sensitivity
NM	normal metabolizer
PM	poor metabolizer
PM+	poor metabolizer with higher enzyme activity than PM
PM*2	PM with two *2 alleles or equivalent (*8, *11, *12)
PM*3	PM with two *3 alleles or equivalent (*4, *5, *6, *13, *14, *15, *25)
PM*2 /*3	PM with one *2 allele or equivalent (*8, *11, *12) and one *3 allele or equivalent (*4, *5, *6, *13, *14, *15, *25)
RM	rapid metabolizer
UM	ultrarapid metabolizer

Abbreviations

bm	best matching information: the information is not available in your language and/or in your country and the best matching translation is used
CPIC	Clinical Pharmacogenetics Implementation Consortium
cs	country-specific: the drug name is not available in your language and/or the drug is not available in your country
CYP	cytochrome P450
DME	drug metabolizing enzyme

 © 2014 - 2021 INTLAB AG

DPWG Dutch Pharmacogenetics Working Group

PGx pharmacogenetics

3.7 Bibliographic references

1 Cacabelos R (ed.) (2012) "Pharmacogenomic Synopsis". In: **World Guide for Drug use and Pharmacogenomics** [DVD-ROM]. La Coruña: EuroEspes Publishing

2 Bristol-Myers Squibb/Sanofi Pharmaceuticals Partnership (2019) **PLAVIX- clopidogrel bisulfate tablet, film coated** [drug label]

3 Brandt JT, Close SL and Iturria SJ et al. (2007) Common polymorphisms of CYP2C19 and CYP2C9 affect the pharmacokinetic and pharmacodynamic response to clopidogrel but not prasugrel. **Journal of Thrombosis and hemostasis**, 5 (12): 2429-2436

4 Hulot JS, Bura A and Villard E et al. (2006) Cytochrome P450 2C19 loss-of-function polymorphism is a major determinant of clopidogrel responsiveness in healthy subjects. **Blood**, 108 (7): 2244-2247

5 Simon T, Verstuyft C and Mary-Krause M et al. (2009) Genetic determinants of response to clopidogrel and cardiovascular events. **New England Journal of Medicine, The**, 360 (4): 363-375

6 Giusti B, Gori AM and Marcucci R et al. (2007) Cytochrome P450 2C19 loss-of-function polymorphism, but not CYP3A4 IVS10 + 12G/A and P2Y12 T744C polymorphisms, is associated with response variability to dual antiplatelet treatment in high-risk vascular patients. **Pharmacogenetics and Genomics**, 17 (12): 1057-1064

7 Fontana P, Senouf D and Mach F (2008) Biological effect of increased maintenance dose of clopidogrel in cardiovascular outpatients and influence of the cytochrome P450 2C19*2 allele on clopidogrel responsiveness. **Thrombosis Reseach**, 121 (4): 463-468

8 Collet JP, Hulot JS and Pena A et al. (2009) Cytochrome P450 2C19 polymorphism in young patients treated with clopidogrel after myocardial infarction: a cohort study. **The Lancet**, 373 (9660): 309-317

9 Mega JL, Close SL and Wiviott SD et al. (2009) Cytochrome p-450 polymorphisms and response to clopidogrel. **The New England Journal of Medicine**, 360 (4): 354-362

10 Sibbing D, Koch W and Gebhard D et al. (2010) Cytochrome 2C19*17 allelic variant, platelet aggregation, bleeding events, and stent thrombosis in clopidogrel-treated patients with coronary stent placement. **Circulation**, 121 (4): 512-518

11 Saydam F, Deirmenci and Birdane A et al. (2017) The CYP2C19*2 and CYP2C19*17 Polymorphisms play a Vital Role in Clopidogrel Responsiveness after Percutaneous Coronary Intervention: A Pharmacogenomics Study. **Basic and Clinical Pharmacology and Toxicology**

12 Scott SA, Sangkuhl K and Stein CM et al. (2013) Clinical Pharmacogenetics Implementation Consortium guidelines for CYP2C19 genotype and clopidogrel therapy: 2013 update [review]. **Clinical Pharmacology and Therapeutics**, 94 (3): 317-323

13 Scott SA, Sangkuhl K and Stein CM et al. (2013) Clinical Pharmacogenetics Implementation Consortium guidelines for CYP2C19 genotype and clopidogrel therapy: 2013 update - Supplemental Material [review]. **Clinical Pharmacology and Therapeutics**: 1-45

14 Simon T and Danchin N (2017) Clinical Impact of Pharmacogenomics of Clopidogrel in Stroke. **Circulation**, 135 (1): 34-37

15 KNMP (2018) **Pharmacogenetic Recommendations November 2018** [online]. Available from: https://www.knmp.nl/@@search?b_start:int=10&SearchableText=pharmacogenetic [Accessed 2019-02-19]

16 Theken KN, Lee CR and Gong L et al. (2020) Clinical Pharmacogenetics Implementation Consortium (CPIC) Guideline for CYP2C9 and Nonsteroidal Anti-inflammatory Drugs - supporting information [review]. **Clinical pharmacology and therapeutics**

17 Chang SY, Li W and Traeger SC et al. (2008) Confirmation that cytochrome P450 2C8 (CYP2C8) plays a minor role in (S)-(+)- and (R)-(-)-ibuprofen hydroxylation in vitro. **Drug metabolism and disposition**, 36 (12): 2513-22

18 Mazaleuskaya LL, Theken KN and Gong L et al. (2015) PharmGKB summary: ibuprofen pathways [review]. **Pharmacogenetics and genomics**, 25 (2): 96-106

19 Ochoa D, Prieto-Pérez R and Román M et al. (2015) Effect of gender and CYP2C9 and CYP2C8 polymorphisms on the pharmacokinetics of ibuprofen enantiomers. **Pharmacogenomics**, 16 (9): 939-48

20 Garcia-Martin E, Martinez C and Tabares B et al. (2004) Interindividual variability in ibuprofen pharmacokinetics is related to interaction of cytochrome P450 2C8 and 2C9 amino acid polymorphisms. **Clinical Pharmacology & Therapeutics**, 76 (2): 119-127

21 Kirchheiner J, Meineke I and Freytag G et al. (2002) Enantiospecific effects of cytochrome P450 2C9 amino acid variants on ibuprofen pharmacokinetics and on the inhibition of cyclooxygenases 1 and 2. **Clinical pharmacology and therapeutics**, 72 (1): 62-75

22 Lopez-Rodriguez R, Novalbos J and Gallego-Sandin S et al. (2008) Influence of CYP2C8 and CYP2C9 Polymorphisms on Pharmacokinetic and Pharmacodynamic Parameters of Racemic and Enantiomeric Forms of Ibuprofen in Healthy Volunteers. **Pharmacological Research**, 58 (1): 77-84

23 Kirchheiner J and Brockmöller J (2005) Clinical Consequences of Cytochrome P450 2C9 Polymorphisms [review]. **Clinical Pharmacology & Therapeutics**, 77 (1): 1-16

24 Theken KN, Lee CR and Gong L et al. (2020) Clinical Pharmacogenetics Implementation Consortium (CPIC) Guideline for CYP2C9 and Nonsteroidal Anti-inflammatory Drugs [review]. **Clinical Pharmacology and Therapeutics**

25 Martínez C, Blanco G and Ladero JM et al. (2004) Genetic predisposition to acute gastrointestinal bleeding after NSAIDs use. **British Journal of Pharmacology**, 141 (2): 205-208

26 Pilotto A, Seripa D and Franceschi M et al. (2007) Genetic susceptibility to nonsteroidal anti-inflammatory drug-related gastroduodenal bleeding: role of cytochrome P450 2C9 polymorphisms. **Gastroenterology**, 133 (2): 465-71

27 Figueiras A, Estany-Gestal A and Aguirre C et al. (2016) CYP2C9 variants as a risk modifier of NSAID-related gastrointestinal bleeding: a case-control study. **Pharmacogenetics and genomics**, 26 (2): 66-73

28 Carbonell N, Verstuyft C and Massard J et al. (2010) CYP2C9*3 Loss-of-Function Allele Is Associated With Acute Upper Gastrointestinal Bleeding Related to the Use of NSAIDs Other Than Aspirin. **Clinical Pharmacology & Therapeutics**, 87 (6): 693-98

29 Pfizer Wyeth Pharmaceuticals Inc (2011) **PROTONIX (pantoprazole sodium)** [drug label]

30 Kuo CH, Lu CY and Shih HY et al. (2014) CYP2C19 polymorphism influences Helicobacter pylori eradication. **World Journal of Gastroenterology**, 20 (43): 16029-16036

31 Furuta T, Sugimoto M and Shirai N (2012) Individualized therapy for gastroesophageal reflux disease: potential impact of pharmacogenetic testing based on CYP2C19 [review]. **Molecular Diagnosis and Therapy**, 16 (4): 223-234

 © 2014 - 2021 INTLAB AG

32 Gawroska-Szklarz B, Siuda A and Kurzawski M et al. (2010) Effects of CYP2C19, MDR1, and interleukin 1-B gene variants on the eradication rate of Helicobacter pylori infection by triple therapy with pantoprazole, amoxicillin, and metronidazole. **European Journal of Clinical Pharmacology**, 66 (7): 681-687

33 Furuta T, Sugimoto M and Shirai N (2013) "Pharmacogenomics of Gastrointestinal Drugs: Focus on Proton Pump Inhibitors". In: Bertino JS, De Vane CL and Fuhr U (eds.) **Pharmacogenomics: An Introduction and Clinical Perspective**. New York: McGraw-Hill. pp. 231-248

34 Jonaitis P, Jonaitis L and Kupcinskas J. et al. (2020) Role of Genetic Polymorphisms of Cytochrome P450 2C19 in Pantoprazole Metabolism and Pantoprazole-based Helicobacter pylori Eradication Regimens. **Current drug metabolism**

35 Gawronska-Szklarz B, Adamiak-Giera U and Wyska E et al. (2012) CYP2C19 polymorphism affects single-dose pharmacokinetics of oral pantoprazole in healthy volunteers. **European Journal of Clinical Pharmacology**, 68 (9): 1267-1274

36 Hagymasi K, Müllner K and Herszenyi L et al. (2011) Update on the pharmacogenomics of proton pump inhibitors [review]. **Pharmacogenomics**, 12 (6): 873-88

37 KNMP (2020) **Pharmacogenetic Recommendations May 2020** [online]. Available from: https://www.knmp.nl [Accessed 2020-06-23]

38 Lima JJ, Thomas CD and Barbarino J et al. (2020) Clinical Pharmacogenetics Implementation Consortium (CPIC) Guideline for CYP2C19 and Proton Pump Inhibitor Dosing [review]. **Clinical Pharmacology and Therapeutics**

39 Lima JJ, Thomas CD and Barbarino J et al. (2020) Clinical Pharmacogenetics Implementation Consortium (CPIC) Guideline for CYP2C19 and Proton Pump Inhibitor Dosing - Supplemental material [review]. **Clinical Pharmacology and Therapeutics**

40 Baxter SD, Teft WA and Choi YH (2014) Tamoxifen-associated hot flash severity is inversely correlated with endoxifen concentration and CYP3A4*22. **Breast Cancer research and treatment**, 145: 419-428

41 Rittweger M and Arasteh K (2007) Clinical pharmacokinetics of darunavir. **Clinical pharmacokinetics**, 46 (9): 739-56

42 Jin Y, Desta Z and Stearns V et al. (2005) CYP2D6 genotype, antidepressant use, and tamoxifen metabolism during adjuvant breast cancer treatment. **Journal of the national cancer institute**, 97 (1): 30-9

43 Mürdter TE, Schroth W and Bacchus-Gerybadze L et al. (2011) Activity levels of tamoxifen metabolites at the estrogen receptor and the impact of genetic polymorphisms of phase I and II enzymes on their concentration levels in plasma. **Clinical pharmacology and therapeutics**, 89 (5): 708-17

44 Teft WA, Gong IY and Dingle B et al. (2013) CYP3A4 and seasonal variation in vitamin D status in addition to CYP2D6 contribute to therapeutic endoxifen level during tamoxifen therapy. **Breast cancer research and treatment**, 139 (1): 95-105

45 Borges S, Desta Z and Li L et al. (2006) Quantitative effect of CYP2D6 genotype and inhibitors on tamoxifen metabolism: implication for optimization of breast cancer treatment. **Clinical pharmacology and therapeutics**, 80 (1): 61-74

46 Lim JS, Chen XA and Singh O et al. (2011) Impact of CYP2D6, CYP3A5, CYP2C9 and CYP2C19 polymorphisms on tamoxifen pharmacokinetics in Asian breast cancer patients. **British journal of clinical pharmacology**, 71 (5): 737-50

47 Madlensky L, Natarajan L and Tchu S et al. (2011) Tamoxifen metabolite concentrations, CYP2D6 genotype, and breast cancer outcomes. **Clinical pharmacology and therapeutics**, 89 (5): 718-25

48 Goetz MP, Suman VJ and Hoskin TL et al. (2013) CYP2D6 metabolism and patient outcome in the Austrian Breast and Colorectal Cancer Study Group trial (ABCSG) 8. **Clinical cancer research**, 19 (2): 500-7

49 Schroth W, Goetz MP and Hamann U et al. (2009) Association between CYP2D6 polymorphisms and outcomes among women with early stage breast cancer treated with tamoxifen. **JAMA**, 302 (13): 1429-36

50 Saladores P, Mürdter T and Eccles D et al. (2015) Tamoxifen metabolism predicts drug concentrations and outcome in premenopausal patients with early breast cancer. **The Pharmacogenomics Journal**, 15 (1): 84-94

51 Goetz MP, Sangkuhl K and Guchelaar HJ et al. (2018) Supplement to: Clinical Pharmacogenetics Implementation Consortium (CPIC) Guideline for CYP2D6 and Tamoxifen Therapy [review]. **Clinical Pharmacology and Therapeutics**, 103 (5)

52 Wigle TJ, Jansen LE and Teft WA et al. (2017) Pharmacogenomics Guided-Personalization of Warfarin and Tamoxifen. **Journal of Personalized Medicine**, 7 (4)

53 Rae JM, Drury S and Hayes DF et al. (2012) CYP2D6 and UGT2B7 genotype and risk of recurrence in tamoxifen-treated breast cancer patients. **Journal of the national cancer institute**, 104 (6): 452-60

54 Regan MM, Leyland-Jones B and Bouzyk M et al. (2012) CYP2D6 genotype and tamoxifen response in postmenopausal women with endocrine-responsive breast cancer: the breast international group 1-98 trial. **Journal of the national cancer institute**, 104 (6): 441-51

55 Johnson JA, Hamadeh IS and Langaee TY (2015) Loss of heterozygosity at the CYP2D6 locus in breast cancer: implications for tamoxifen pharmacogenetic studies [review]. **Journal of the national cancer institute**, 107 (2): dju437

56 Province MA, Goetz MP and Brauch H et al. (2014) CYP2D6 genotype and adjuvant tamoxifen: meta-analysis of heterogeneous study populations. **Clinical pharmacology and therapeutics**, 95 (2): 216-27

57 Irvin WJ Jr, Walko CM and Weck KE et al. (2011) Genotype-guided tamoxifen dosing increases active metabolite exposure in women with reduced CYP2D6 metabolism: a multicenter study. **Journal of Clinical Oncology**, 29 (24): 3232-9

58 Hertz DL, Deal A and Ibrahim JG et al. (2016) Tamoxifen Dose Escalation in Patients With Diminished CYP2D6 Activity Normalizes Endoxifen Concentrations Without Increasing Toxicity. **The Oncologist**, 21 (7): 795-803

59 Dezentjé VO, Opdam FL and Gelderblom H et al. (2015) CYP2D6 genotype- and endoxifen-guided tamoxifen dose escalation increases endoxifen serum concentrations without increasing side effects. **Breast Cancer Research and Treatment**, 153 (3): 583-90

60 Goetz MP, Sangkuhl K and Guchelaar HJ et al. (2018) Clinical Pharmacogenetics Implementation Consortium (CPIC) Guideline for CYP2D6 and Tamoxifen Therapy. **Clinical Pharmacology and Therapeutics**, 103 (5): 770-777

61 Early Breast Cancer Trialists' Collaborative Group (2015) Aromatase inhibitors versus tamoxifen in early breast cancer: patient-level meta-analysis of the randomised trials [review]. **Lancet**, 386 (10001): 1341-1352

62 Swen JJ, Nijenhuis M and de Boer A et al. (2011) Pharmacogenetics: from bench to byte - an update of guidelines. **Clinical Pharmacology and Therapeutics**, 89: 662-673

63 PharmGKB **tamoxifen dosing guidelines** [online]. Available from: https://www.pharmgkb.org/chemical/PA451581#tabview=tab0&subtab=31 [Accessed 2016-02-22]

64 Gillen C, Haurand M and Kobelt DJ et al. (2000) Affinity, potency and efficacy of tramadol and its metabolites at the cloned human mu-opioid receptor. **Naunyn-Schmiedeberg's Archives of Pharmacology**, 2: 116-121

65 Lee S-J, Lee S-S and Shin J-G (2013) "Pharmacogenetics of Cytochrome P450". In: Bertino JS, De Bane CL and Fuhr U (eds.) **Pharmacogenomics: An Introduction and Clinical Perspective**. New York: McGraw-Hill.

 © 2014 - 2021 INTLAB AG

66 Stamer UM, Musshoff F and Kobilay M et al. (2007) Concentrations of tramadol and O-desmethyltramadol enantiomers in different CYP2D6 genotypes. **Clinical Pharmacology and Therapeutics**, 82 (1): 41-47

67 Pedersen RS, Damkier P and Brøsen K (2006) Enantioselective pharmacokinetics of tramadol in CYP2D6 extensive and poor metabolizers. **European Journal of Clinical Pharmacology**, 62 (7): 513-521

68 García-Quetglas E, Azanza JR and Sádaba B et al. (2007) Pharmacokinetics of tramadol enantiomers and their respective phase I metabolites in relation to CYP2D6 phenotype. **Pharmacological Research**, 55 (2): 122-130

69 Gan SH, Ismail R and Wan Adnan WA et al. (2007) Impact of CYP2D6 genetic polymorphism on tramadol pharmacokinetics and pharmacodynamics.. **Molecular Diagnosis and Therapy**, 11 (3): 171-181

70 PharmGKB **Drug Label Information and Legend** [online]. Available from: https://www.pharmgkb.org/page/drugLabelLegend [Accessed 2018-04-17]

71 Swen JJ, Wilting I and de Goede AL et al. (2008) Pharmacogenetics: from bench to byte. **Clinical Pharmacology and Therapeutics**, 83 (5): 781-787

72 Whirl-Carrillo M, McDonagh EM and Hebert JM et al. (2012) Pharmacogenomics knowledge for personalized medicine [review]. **Clinical Pharmacology & Therapeutics**, 92 (4): 414-7

Laboratory

Laboratory analysis was carried out under Swiss law (GUMG) by:

labormedizinisches zentrum Dr Risch AG
Waldeggstrasse 37
CH-3097 Liebefeld
Telephone: +41 58 523 34 60
E-mail: genetik@risch.ch
Website: http://www.risch.ch

Version

Software: 1.9.0-0

Manufacturer

This report was generated by SONOGEN XP, an *in vitro* diagnostic medical device, manufactured by:

INTLAB AG
Seefeldstrasse 214
CH-8008 Zürich
Telephone: +41 43 508 69 36
E-mail: support@sonogen.eu
Website: http://www.sonogen.eu

 © 2014 - 2021 INTLAB AG

SONOGEN

laboormedizinisches zentrum
centre des laboratoires médicaux
centro medicina di laboratorio
Dr Risch

SONOGEN XP report for Annemarie-Clara Muster - brief version

**First name:**	Annemarie-Clara	**Laboratory sample ID:**	12345
**Last name:**	Muster	**Sample collection date:**	October 22, 2020
**Date of birth:**	April 17, 1975	**Report date:**	March 1, 2021
**Gender:**	female		
**Treatment:**	clopidogrel, ibuprofen, pantoprazole, pregabalin, tamoxifen, tramadol		

PGx profile

Gene	Genotype	Predicted phenotype	Effect
CYP2C9	*3/*3	PM*3	very slow metabolism
CYP2C19	*1/*3	IM	slow metabolism
CYP2D6	*4J/*10	IM	slow metabolism
DPYD	*1/HapB3	IM+	slow metabolism
POR	*28/*28	increased function	fast metabolism
VKORC1	-1639GA	decreased function	increased drug efficacy

The tested markers show normal genotypes and/or phenotypes for:
ABCB1, COMT, CYP1A2, CYP2B6, CYP3A4, CYP3A5, CYP4F2, OPRM1, SLCO1B1, TPMT

Drug - PGx interactions of current treatment

	Normal risk	Use with caution	High risk
**clopidogrel**<br>CYP2C19 IM			• **Choose alternative antiplatelet therapy** if no contraindication (e.g., prasugrel, ticagrelor).
**ibuprofen**<br>CYP2C9 PM*3		• **Initiate with 25-50% of lowest starting dose and titrate dose upward to clinical effect or 25-50% of maximum dose.**<br>• **Carefully monitor adverse events or**<br>• **Consider an alternate therapy not metabolized by CYP2C9 or not significantly impacted by CYP2C9 genetic variants.**	
**pantoprazole**<br>CYP2C19 IM		• Initiate standard starting daily dose.<br>• For chronic therapy (>12 weeks) and once efficacy achieved, **consider 50% reduction in daily dose**<br>• **Monitor for continued efficacy.**	
**tamoxifen**<br>CYP2D6 IM			• **Consider alternative hormonal therapy** such as aromatase inhibitor for postmenopausal women or aromatase inhibitor along with ovarian function suppression in premenopausal women.<br>• **If aromatase inhibitor use is contraindicated, consider use of a higher tamoxifen dose (40 mg/day).**<br>• **Avoid concomitant use of CYP2D6 inhibitors** (strong to weak)**.**
**tramadol**<br>CYP2D6 IM		• **Be alert to decreased efficacy** (symptoms of insufficient pain relief).<br>• **Consider dose increase.**<br>• **If response is still inadequate, select alternative drug**- not oxycodone or codeine-	

Current literature (e.g. dosing guidelines, drug labels, peer reviewed articles) does not allow PGx-based recommendation for: pregabalin

© 2014 - 2021 INTLAB AG

Predictable drug - PGx interactions

Table shows potential interactions of specific drugs with patient’s PGx profile. These drugs are related to biomarkers, for which drug label recommendations or dosing guidelines exist, or for which LoE is at least C. For suggested action and detailed information, please indicate drug of interest in patient’s treatment and refer to SONOGEN detailed report or consult drug labels or dosing guidelines.

Normal risk	Use with caution	High risk
atorvastatin (1)	amitriptyline (2)	acenocoumarol (1)
azathioprine (3)	aripiprazole (2)	capecitabine (2)
brexpiprazole (2)	atomoxetine (2)	clopidogrel (2)
carvedilol (2)	carisoprodol (2)	flecainide (1)
clozapine (2)	celecoxib (2)	fluorouracil (2)
darifenacin (2)	cevimeline (2)	phenprocoumon (1)
efavirenz (2)	citalopram (2)	phenytoin (2)
fesoterodine (2)	clobazam (2)	propafenone (2)
fluvoxamine (1)	clomipramine (2)	tamoxifen (1)
haloperidol (1)	codeine (2)	warfarin (2)
mercaptopurine (3)	desipramine (2)	
mirtazapine (1)	diclofenac (1)	
morphine (1)	doxepin (2)	
nelfinavir (1)	escitalopram (2)	
olanzapine (1)	flurbiprofen (2)	
ondansetron (1)	glyburide (2)	
paroxetine (1)	ibuprofen (1)	
propofol (1)	iloperidone (2)	
rosuvastatin (2)	imipramine (2)	
sertraline (1)	lansoprazole (1)	
simvastatin (1)	lornoxicam (1)	
tacrolimus (1)	meloxicam (2)	
tioguanine (3)	metoprolol (1)	
tropisetron[cs] (1)	nortriptyline (2)	
vortioxetine (2)	omeprazole (2)	
	oxycodone (1)	
	pantoprazole (2)	
	perphenazine (2)	
	pimozide (4)	
	piroxicam (2)	
	risperidone (1)	
	siponimod (4)	
	tak-390mr (2)	
	tenoxicam (1)	
	tetrabenazine (4)	
	thioridazine (2)	
	tramadol (2)	
	trimipramine (2)	
	venlafaxine (2)	
	voriconazole (2)	
	zuclopenthixol (1)	

() PGx information included in the drug label; based on Pharmacogenomics Knowledgebase (PharmGKB) and classified into the following categories: (4) required, (3) recommended, (2) actionable, (1) informative

Disclaimer

The present individual treatment optimization proposal and the related information was generated by SONOGEN XP - a clinical decision support and pharmacogenetic expert system. This software is an in vitro medical device and has been developed according to the directive on in vitro diagnostic medical devices (Directive 98/79/EC of the European Parliament and of the Council). The containing information has been collected and reviewed to our best knowledge, however there is no guarantee that it contains the latest scientific findings and that all adverse or important outcomes will be reported in the literature and integrated in the SONOGEN XP software. The responsibility for a correct drug-treatment prescription lies with the treating physician and the user should always apply his independent professional judgement.

Limitation

This pharmacogenetic test will not detect all the known mutations of a gene. Absence of a detectable gene mutation does not rule out the possibility of an altered phenotype due to the presence of an undetected mutation or due to other factors influencing the drug efficacy, such as drug-drug-interactions, comorbidities or lifestyle habits.

For further information, please refer to the detailed report.

Software version: 1.9.0-0

© 2014 - 2021 INTLAB AG

INTLAB AG, Seefeldstrasse 214, CH-8008 Zürich,
+41 43 508 69 36, support@sonogen.eu,
http://www.sonogen.eu

labormedizinisches zentrum Dr Risch AG, Waldeggstrasse 37,
CH-3097 Liebefeld, +41 58 523 34 60, genetik@risch.ch,
http://www.risch.ch

© 2014 - 2021 INTLAB AG

SONOGEN XP report for Annemarie-Clara Muster - PGx profile explanation

**First name:**	Annemarie-Clara	**Laboratory sample ID:**	12345
**Last name:**	Muster	**Sample collection date:**	October 22, 2020
**Date of birth:**	April 17, 1975	**Report date:**	March 1, 2021
**Gender:**	female		

1 Introduction

Pharmacogenetics is the study of variations in genes that code for drug metabolizing enzymes, drug transporters, drug targets, or proteins involved in the immune response. These variants are associated with a variable response or reaction to a large amount of medications. The knowledge of these variants in a patient (PGx profile) helps to increase drug efficacy and tolerability and individualize a patient's treatment.

Phase I drug metabolizing enzymes (DME1) catalyze the first step in the metabolism of many drugs. Genetic polymorphisms are responsible for a variability in the expression and activity of most DMEs. This can lead to interindividual differences in the metabolism of drugs and may therefore influence the drug plasma levels and the drug response. [1]

Polymorphisms in the cytochrome P450 (CYP) family account for the most frequent variations in DMEs. Nearly 80% of all drugs used today are metabolized by one or more P450 enzymes. The majority of the P450 enzymes are expressed in the liver, but some are also found in other tissues such as gastrointestinal tract, central nervous system or lung. Some CYP genes are highly polymorphic and contribute significantly to adverse drug reactions and therapeutic failures. [1 2]

Individuals can be categorized into phenotypic groups according to their metabolic rate: [1 3]

PM: Poor metabolizers have no or drastically reduced amount of functional enzymes. They have no or very reduced metabolism of substrate drugs leading to increased plasma concentrations of these drugs with a higher risk of adverse effects.

IM: Intermediate metabolizers have a decreased enzymatic activity. They metabolize substrate drugs more slowly and may have increased plasma concentrations of these drugs.

NM: Normal metabolizers have normal enzymatic activity and normal drug metabolism.

RM: Rapid metabolizers have an increased enzymatic activity. They metabolize substrate drugs more rapidly and may have reduced plasma concentrations of these drugs.

UM: Ultrarapid metabolizers have a strongly increased enzymatic activity. They metabolize substrate drugs more rapidly and have reduced plasma concentrations of these drugs with a higher risk of therapeutic failure.

The rate of metabolism for a certain drug can differ 1000-fold between the PMs and UMs. Such Patients may require dose adjustments or the choice of an alternative drug. [1] In the case of prodrugs this effect will be inversed, meaning lower concentrations of the active ingredient in PMs and higher concentrations in UMs.

The alleles are defined according to the nomenclature for cytochromes. [4] The *1 allele is the wild-type allele with normal function and is given in the absence of any known variants.

The phase II drug metabolizing enzymes (DME2) facilitate the elimination of endogenous and foreign compounds. Many substrates are first activated by phase I enzymes and then further metabolized by phase II enzymes. Some of the phase II enzymes, including TPMT, DPYD and COMT, are highly polymorphic and show interindividual differences in drug response. [5]

Drug Transporters, e.g. SLCO1B1 and ABCB1, which are highly expressed in organs such as the liver, intestine and kidney or blood brain barrier, seem to play a major role in drug disposition and efficacy. [6]

© 2014 - 2021 INTLAB AG

2 PGx profile

Gene	Genotype	Predicted phenotype	Effect	Tested alleles
CYP2C9	*3/*3	PM*3	very slow metabolism	*1, *2, *3, *4, *5, *6, *8, *11, *12, *13, *15, *25, *27
CYP2C19	*1/*3	IM	slow metabolism	*1, *2, *3, *4A, *4B, *5, *6, *7, *8, *17
CYP2D6	*4J/*10	IM	slow metabolism	*1, *2, *3, *4, *4J, *4K, *4M, *5, *6, *6C, *7, *8, *9, *10, *11, *12, *14A, *14B, *15, *18, *19, *20, *29, *34, *39, *41, *69, CNV
DPYD	*1/HapB3	IM+	slow metabolism	*1, *2A, *13, 2846T, HapB3
POR	*28/*28	increased function	fast metabolism	*1, *28
VKORC1	-1639GA	decreased function	increased drug efficacy	-1639A, -1639G
ABCB1	1236CC, 2677GG, 3435CC	CGC/CGC	normal drug efficacy	CAC, CAT, CGC, CGT, CTC, CTT, TAC, TAT, TGC, TGT, TTC, TTT
COMT	High /Intermediate	APS	normal metabolism	High, Intermediate, Low
CYP1A2	*1A/*1A	NM	normal metabolism	*1A, *1C, *1F, *1K, *1L, *7, *11
CYP2B6	*1/*1	NM	normal metabolism	*1, *6, *18
CYP3A4	*1/*1	*22 non-carrier	normal metabolism	*1, *2, *17, *22
CYP3A5	*3/*3	non-expresser	normal metabolism	*1, *2, *3, *3+2, *6, *7
CYP4F2	*1/*1	NM	normal metabolism	*1, *3
OPRM1	118AA	normal function	normal drug efficacy	118A, 118G
SLCO1B1	*1a/*1a	normal function	normal drug efficacy	*1a, *5
TPMT	*1/*1	NM	normal metabolism	*1, *2, *3A, *3B, *3C, *4

3 Explanation of PGx profile

3.1 ABCB1 1236CC, 2677GG, 3435CC - CGC/CGC

ATP binding cassette transporter (ABCB1), also called P-gp (P-glycoprotein) or MDR1 (multidrug resistance) facilitate the export of a variety of compounds including drugs, such as anticancer agents, antidepressants, antibiotics, immunosuppressants or cardiac drugs. It is expressed in tissues such as intestine, liver, kidney and the blood-brain-barrier, influencing intestinal drug absorption and limiting oral bioavailability. A variety of polymorphisms has been identified but the effects on ABCB1 function are complex and difficult to interpret as there are no phenotypes defined, rather haplotypes. [6] [7] [8] [9]
The genoyping procedure does not permit to define the exact haplotype sequence of a given allele and the most likely combination, based on statistical results, is therefore preferentially chosen. In this context, it should be noted that the haplotype frequency for the positions 1236-2677-3435 significantly differs between ethnicities. While the dominant haplotypes for Caucasians are TTT and CGC, the majority of African-Americans have CGC, and in the Japanese population three (CAC, CGC, and TTT) common haplotypes were described. [6] [10]

C at position 1236 is the reference allele.
G at position 2677 is the normal activity allele.
C at position 3435 is the normal activity allele.

Haplotype CGC is the reference sequence or *1. [11] [12]

3.2 COMT High/Intermediate - APS

Catechol-O-Methyltransferase (COMT) is an enzyme that inactivates catecholamines, particularly dopamine. COMT regulates cognitive function, memory, mood and pain perception. A variety of drugs, such as opioids, SSRIs and antipsychotics, may be directly or indirectly impacted by variants of COMT. [13] [14] [15] [16] [17]

 © 2014 - 2021 INTLAB AG

High COMT activity with the haplotype GCGG (1-98G, 186C, 408G, 472G) at rs6269, rs4633, rs4818 and rs4680. [18] [16]
Intermediate COMT activity with the haplotype ATCA (1-98A, 186T, 408C, 472A) at rs6269, rs4633, rs4818 and rs4680. [18] [16]

Intermediate COMT activity was associated with intermediate or average pain sensitivity (APS). [18] [19]

3.3 CYP1A2 *1A/*1A - NM

CYP1A2 is responsible for the metabolism of approximately 9% of the prescription drugs, including analgesics, antipsychotics, antidepressants and cardiovascular drugs, but also caffeine. The expression of CYP1A2 is highly inducible by other drugs (e.g. carbamazepine, omeprazole or primaquine) and environmental factors such as cigarette smoke. [1] [2]

*1A is defined as the wildtype allele with normal function. [20]

NMs have a normal enzyme activity with a normal metabolism of CYP1A2 substrates.

3.4 CYP2B6 *1/*1 - NM

CYP2B6 is involved in the metabolism of many clinically important drugs, such as ketamine, propofol, bupropion and HIV reverse transcriptase inhibitors and in the activation of cytotoxic prodrugs, such as cyclophosphamide. Currently, over 38 alleles were identified for CYP2B6. The expression level of the enzyme in the liver varies 20-250-fold between individuals. CYP2B6 is highly inducible by many drugs. [1] [21]

*1 allele is defined as the wildtype allele with normal function. [22]

NMs have a normal enzyme activity with a normal metabolism of CYP2B6 substrates.

3.5 CYP2C9 *3/*3 - PM*3

CYP2C9 is the major enzyme found in the human liver and participates in the metabolism of about 10-20% of commonly prescribed drugs, including anticoagulants, antidiabetic agents, antiepileptics, and nonsteroidal anti-inflammatory drugs. [1]
CYP2C9 is highly polymorphic with about 60 different alleles described. [23] These variations have been recognized to be in part responsible for ADRs, as many substrate drugs of CYP2C9 have a narrow therapeutic index. [2]

*3 is a variant allele with drastically decreased function. [23] [2]

PM*3 have two **3* alleles or equivalent (**4, *5, *6, *13, *14, *15, *25*). PMs have a drastically reduced enzyme activity and a slow metabolism of the substrates. This may lead to higher concentrations of the drugs and an increased risk of ADRs. The frequency of CYP2C9 PMs is relatively low, but the clinical consequences can be serious with severe and life-threatening ADRs. [24] [2] This includes, for example, hypoglycaemia with antidiabetic drugs, gastrointestinal bleeding as a result from treatment with NSAIDs and serious bleedings with anticoagulants. [2]

3.6 CYP2C19 *1/*3 - IM

CYP2C19 is involved in the metabolism of about 7% of all drugs, such as antidepressants, proton pump inhibitors, antiepileptics and the anticoagulant clopidogrel. [1] [2] CYP2C19 has a relative high number and frequency of non-functional alleles. [25] [2]

*1 allele is defined as the wildtype allele with normal function. [25]
*3 is a non-functional allele. [25]

IMs have a reduced enzyme activity which may cause higher plasma concentrations of CYP2C19 substrate drugs. [2] For prodrugs (e.g., clopidogrel), the effect is inversed with lower plasma concentrations of the active metabolite.

3.7 CYP2D6 *4J/*10 - IM

CYP2D6 is involved in the oxidation of 20-25% of all drugs in clinical use from virtually all therapeutic classes, like antiarrhythmics, tricyclic and second generation antidepressants, antipsychotics, beta-blockers, opioids, as well as anti-cancer drugs. [2]
Until now, there are more than 100 different alleles described for CYP2D6. [26]

*4J is a non-functional allele. [26]
*10 is a variant allele with decreased function. [27]

IMs have a reduced enzyme activity which may lead to higher concentrations of CYP2D6 substrate drugs. For prodrugs (e.g. codeine, tramadol), the effect is inversed with lower plasma concentrations of the active metabolite.

3.8 CYP3A4 *1/*1 - *22 non-carrier

CYP3A enzymes (CYP3A4 and CYP3A5) have similar substrate specificities and are involved in the metabolism of 50% of all currently used drugs. Substrates of CYP3A4 include immunosuppressants, antibiotics and anticancer drugs. The activity of CYP3A4 is characterized by widespread variation, but so far, only one allele (*22) was found to influence the enzyme expression and function. Genetic factors account for 70-90% of the CYP3A variability in substrate clearance, but environmental or endogenous factors may lead to even greater level of variability due to induction or inhibition of enzyme activity. [1] [28] [2]

*1 allele is defined as the wildtype allele with normal function. [29]

© 2014 - 2021 INTLAB AG

CYP3A4*22 non-carriers have a normal enzyme activity with a normal metabolism of CYP3A4 substrates.

3.9 CYP3A5 *3/*3 - non-expresser

CYP3A5 is only expressed in a limited number of individuals. As the non-functional alleles *3, *6, *7 occur with higher frequencies than the functional *1 allele, the CYP3A5 non-expresser with no or nearly no enzyme activity is the normal phenotype. [1] [2]

*3 is a frequent variant allele with severely decreased enzyme function. [30]

CYP3A5 non-expressers have no or drastically reduced expression of functional enzyme. This is considered as the normal phenotype. [1]

3.10 CYP4F2 *1/*1 - NM

CYP4F2 contributes to vitamin K1 oxidation and thus inactivation. The CYP4F2 V433M polymorphism (rs2108622) results in decreased protein levels and thus in decreased vitamin K1 oxidation leading to increased coumarin dose requirement. [31] [32] [33] [34]

*1 is defined as the wildtype allele with normal function. [31]

NMs are associated with normal amount of CYP4F2 protein and no effect on coumarin sensitivity. [31] [34] [32]

3.11 DPYD *1/HapB3 - IM+

The enzyme dihydropyrimidine dehydrogenase (DPD) is encoded by the DPYD gene and is critical in the metabolism of pyrimidine drugs, such as 5-fluorouracil, capecitabine or tegafur. The DPD activity can vary up to 20-fold due to genetic variations. [7]

*1 allele is defined as the wildtype allele with normal enzyme function. [35]
HapB3 (haplotype B3) is a variant with decreased enzyme function. [36] [37]

IM+ have a reduced DPD activity. This leads to an increased risk of severe toxicity with pyrimidine drugs and a dose reduction is recommended. [38] [36]

3.12 OPRM1 118AA - normal function

The mu-opioid receptor 1 (OPRM1) is the primary site of action for opioid analgesics, including morphine and fentanyl. The OPRM1 gene is highly polymorphic. One of the more frequent polymorphism is 118 A>G, which alters the receptor expression. [39] [40]

A at position 118 is the wildtype allele.

The normal function phenotype is associated with normal OPRM1 expression and function. [40]

3.13 POR *28/*28 - increased function

POR (P450 oxidoreductase) is required for the electron transfer from NADPH to CYP to assure the enzymatic function. Therefore, functional variants of POR can have an impact on the activity of CYP enzymes.
POR is highly polymorphic and more than 40 variant alleles have been described so far. The variant *POR*28* (rs1057868; A503V) has an increased activity of POR and was associated with increased CYP3A4 and 3A5 activities. [41] [42]

*28 is a common variant allele with increased POR activity. [42] [41]

The increased function phenotype shows an increased POR activity and is associated with increased activity of CYP3A4 and CYP3A5. [41]
This leads to a higher dose requirement of tacrolimus in CYP3A5 expressers. [43] [44] [45] [42] [46]

3.14 SLCO1B1 *1a/*1a - normal function

Solute carrier organic anion transporter (SLCO1B1) or OATP1B1 (organic anion transporting polypeptide) is a hepatic uptake transporter which acts as a rate limiting step in drug elimination, such as statins or rifampin. Genetic variations can cause an impaired SLCO1B1 function and a reduced elimination of the substrate drugs. [7]

*1a allele is defined as the wildtype allele with normal enzyme function.

The normal function phenotype is associated with a normal transport of SLCO1B1 substrates.

3.15 TPMT *1/*1 - NM

Thiopurine-S-methyltransferase (TPMT) metabolizes thiopurines (e.g. mercaptopurine, azathioprine, thioguanine) to inactive metabolites, preventing the conversion into toxic thioguanine nucleotides. Patients with reduced TPMT activity have an increased risk of side effects, such as myelosuppression. [5] [47]

*1 allele is defined as the wildtype allele with normal enzyme function. [48]

NMs have a normal enzyme activity with a normal metabolism of thiopurines.

 © 2014 - 2021 INTLAB AG

3.16 VKORC1 -1639GA - decreased function

Coumarin anticoagulants (e.g. warfarin, acenocoumarol, phenprocoumon) act by inhibiting the enzyme vitamin K epoxide reductase complex 1 (VKORC1). The polymorphism -1639G>A has been shown to affect the levels of *VKORC1* gene expression and therefore the amount of protein and the required dose of coumarin anticoagulants. [49]

A at position -1639 is a variant allele with lower amount of VKORC1. [49]
G at position -1639 is defined as the wildtype allele with normal amount of VKORC1. [49]

The decreased function phehotpye is associated with reduced amount of VKORC1 protein and an increased coumarin sensitivity. [49] [50]

3.17 CYP2C9 *3/*3 - PM*3 and CYP4F2 *1/*1 - NM and VKORC1 -1639GA - decreased function

High coumarin sensitivity
The contribution of VKORC1 to the variation in dose requirement of coumarins is larger (approximately 30%) than the contribution of CYP2C9 (less than 12%) and CYP4F2 (1% - 5%). CYP2C9 plays a less important role in phenprocoumon metabolism compared to warfarin or acenocoumarol. Together with non-genetic factors, 50-60% of the variability can be predicted. [1] [51] [52] [34] [33] [53]

High coumarin sensitivity means very low dose requirement of coumarins. [50] Dose adjustment is more complex and the time to reach stable target INR is much longer. [54] The use of an alternative drug (non-coumarin drug) should be considered.

4 Limitation

This pharmacogenetic test will not detect all the known mutations of a gene. Absence of a detectable gene mutation does not rule out the possibility of an altered phenotype due to the presence of an undetected mutation or due to other factors influencing the drug efficacy, such as drug-drug-interactions, comorbidities or lifestyle habits.

5 Bibliographic references

1. Dolzan V (2012) "Pharmacogenetics in Drug Metabolism: Role of Phase I Enzymes". In: Maitland-van der Zee A-H and Daly AK (eds.) **Pharmacogenetics and Individualized Therapy**. Hoboken: Wiley.
2. Zanger UM and Schwab M (2013) Cytochrome P450 enzymes in drug metabolism: Regulation of gene expression, enzyme activities, and impact of genetic variation [review]. **Pharmacology & Therapeutics**, 138 (1): 103-141
3. Samer CF, Lorenzini KI and Rollason V et al. (2013) Applications of CYP450 Testing in the Clinical Setting. **Molecular Diagnosis & Therapy**, 17: 165-184
4. Sim SC and Ingelman-Sundberg M (2010) The Human Cytochrome P450 (CYP) Allele Nomenclature website: a peer-reviewed database of CYP variants and their associated effects. **Human Genomics**, 4 (4): 278-81
5. Cascorbi I (2012) "Pharmacogenetics of Phase II Drug Metabolizing Enzymes". In: Maitland-van der Zee A-H and Daly AK (eds.) **Pharmacogenetics and Individualized Therapy**. Hoboken: Wiley.
6. Meyer zu Schwabedissen HE, Grube M and Koemer HK (2012) "Pharmacogenetics of Drug Tranporters". In: Maitland-van der Zee A-H and Daly AK (eds.) **Pharmacogenetics and Individualized Therapy**. Hoboken: Wiley. pp. 101-148
7. Alsanosi SMM, Skiffington C and Padmanabhan S (2014) "Pharmacokinetic Pharmacogenomics". In: Padmanabhan S (ed.) **Handbook of pharmacogenomics and stratified medicine**. London: Academic Press. pp. 341-364
8. de Klerk OL, Nolte IM and Bet PM et al. (2013) ABCB1 gene variants influence tolerance to selective serotonin reuptake inhibitors in a large sample of Dutch cases with major depressive disorder. **Pharmacogenomics Journal, The**, 13 (4): 349-353
9. Breitenstein B, Scheuer S and Pfister H et al. (2014) The clinical application of ABCB1 genotyping in antidepressant treatment: a pilot study. **CNS Spectrums**, 19 (2): 165-175
10. Fung KL and Gottesman MM (2009) A synonymous polymorphism in a common MDR1 (ABCB1) haplotype shapes protein function. **Biochimica et Biophysica Acta**, 1794 (5): 860-71
11. Kim RB, Leake BF and Choo EF et al. (2001) Identification of functionally variant MDR1 alleles among European Americans and African Americans. **Clinical Pharmacology and Therapeutics**, 70 (2): 189-199
12. Tsunoda SM, Bednarczyk D and Okochi H (2013) "Drug Transporters". In: Bertino JS, De Vane CL and Fuhr U et al. (eds.) **Pharmacogenomics: An Introduction and Clinical Perspective**. New York: McGraw-Hill. pp. 89-104
13. Tammimäki A and Männistö PT (2012) Catechol-O-methyltransferase gene polymorphism and chronic human pain: a systematic review and meta-analysis. **Pharmacogenetics and genomics**, 22 (9): 637-91
14. De Gregori M, Garbin G and De Gregori S et al. (2013) Genetic variability at COMT but not at OPRM1 and UGT2B7 loci modulates morphine analgesic response in acute postoperative pain. **European journal of clinical pharmacology**, 69 (9): 1651-8
15. Nackley AG, Shabalina SA and Lambert JE et al. (2009) Low enzymatic activity haplotypes of the human catechol-O-methyltransferase gene: enrichment for marker SNPs. **PLoS One**, 4 (4): e5237
16. Nackley AG, Shabalina SA and Tchivileva IE (2006) Human catechol-O-methyltransferase haplotypes modulate protein expression by altering mRNA secondary structure. **Science**, 314 (5807): 1930-3
17. Reyes-Gibby CC, Shete S and Rakvag T et al. (2007) Exploring joint effects of genes and the clinical efficacy of morphine for cancer pain: OPRM1 and COMT gene. **Pain**, 130 (1-2): 25-30
18. Diatchenko L, Slade GD and Nackley AG et al. (2005) Genetic basis for individual variations in pain perception and the development of a chronic pain condition. **Human molecular genetics**, 14 (1): 135-43
19. Diatchenko L, Nackley AG and Slade GD et al. (2006) Catechol-O-methyltransferase gene polymorphisms are associated with multiple pain-evoking stimuli. **Pain**, 125 (3): 216-24

 © 2014 - 2021 INTLAB AG

20 The Human Cytochrome P450 (CYP) Allele Nomenclature Database **CYP1A2 allele nomenclature** [online]. Available from: http://www.cypalleles.ki.se/cyp1a2.htm [Accessed 2016-05-06]

21 Zanger UM and Klein K (2013) Pharmacogenetics of cytochrome P450 2B6 (CYP2B6): advances on polymorphisms, mechanisms, and clinical relevance. **Frontiers in Genetics**, 4 (24)

22 PharmVar **CYP2B6** [online]. Available from: https://www.pharmvar.org/gene/CYP2B6 [Accessed 2019-09-09]

23 PharmVar - Pharmacogene Variation Consortium **CYP2C9** [online]. Available from: https://www.pharmvar.org/gene/CYP2C9 [Accessed 2018-10-29]

24 Goldstein JA (2001) Clinical relevance of genetic polymorphisms in the human CYP2C subfamily [review]. **British Journal of Clinical Pharmacology**, 52 (4): 349-355

25 PharmVar - Pharmacogene Variation Consortium **CYP2C19** [online]. Available from: https://www.pharmvar.org/gene/CYP2C19 [Accessed 2018-10-18]

26 PharmVar - Pharmacogene Variation Consortium **CYP2D6** [online]. Available from: https://www.pharmvar.org/gene/CYP2D6 [Accessed 2019-03-08]

27 The Human Cytochrome P450 (CYP) Allele Nomenclature Database **CYP2D6 allele nomenclature** [online]. Available from: http://www.cypalleles.ki.se/cyp2d6.htm [Accessed 2016-05-04]

28 Elens L, Van Gelder T and Hesselink DA et al. (2013) CYP3A4*22: promising newly identified CYP3A4 variant allele for personalizing pharmacotherapy [review]. **Pharmacogenomics**, 14 (1): 47-62

29 The Human Cytochrome P450 (CYP) Allele Nomenclature Database **CYP3A4 allele nomenclature** [online]. Available from: http://www.cypalleles.ki.se/cyp3a4.htm [Accessed 2016-05-06]

30 The Human Cytochrome P450 (CYP) Allele Nomenclature Database **CYP3A5 allele nomenclature** [online]. Available from: http://www.cypalleles.ki.se/cyp3a5.htm [Accessed 2014-12-22]

31 Alvarellos ML, Sangkuhl K and Daneshjou R et al. (2015) PharmGKB summary: very important pharmacogene information for CYP4F2 [review]. **Pharmacogenetics and Genomics**, 25 (1): 41-7

32 Danese E, Montagnana M and Johnson JA et al. (2012) Impact of the CYP4F2 p.V433M polymorphism on coumarin dose requirement: systematic review and meta-analysis [review]. **Clinical Pharmacology and Therapeutics**, 92 (6): 746-56

33 Daly AK (2013) Optimal dosing of warfarin and other coumarin anticoagulants: the role of genetic polymorphisms [review]. **Archives of Toxicology** , 87 (3): 407-20

34 Teichert M, Eijgelsheim M and Uitterlinden AG et al. (2011) Dependency of phenprocoumon dosage on polymorphisms in the VKORC1, CYP2C9, and CYP4F2 genes. **Pharmacogenetics and Genomics**, 21 (1): 26-34

35 McLeod HL, Collie-Duguid ES and Vreken P et al. (1998) Nomenclature for human DPYD alleles. **Pharmacogenetics**, 8 (6): 455-9

36 Amstutz U, Henricks LM and Offer SM et al. (2018) Clinical Pharmacogenetics Implementation Consortium (CPIC) Guideline for Dihydropyrimidine Dehydrogenase Genotype and Fluoropyrimidine Dosing: 2017 Update. **Clinical Pharmacology and Therapeutics**, 103 (2): 210-216

37 Henricks LM, Lunenburg CA and Meulendijks D et al. (2015) Translating DPYD genotype into DPD phenotype: using the DPYD gene activity score. **Pharmacogenomics**, 16 (11): 1277-86

38 Caudle KE, Thorn CF and Klein TE et al. (2013) Clinical Pharmacogenetics Implementation Consortium Guidelines for Dihydropyrimidine Dehydrogenase Genotype and Fluoropyrimidine Dosing [review]. **Clinical Pharmacology and Therapeutics**, 94 (6): 640-645

39 Lötsch J (2012) "Pharmacogenetics of Pain Medication". In: Maitland-van der Zee A-H and Daly AK (eds.) **Pharmacogenetics and Individualized Therapy**. Hoboken: Wiley.

40 OMIM **OMIM 600018: OPIOID RECEPTOR, MU-1; OPRM1** [online]. Available from: http://omim.org/entry/600018 [Accessed 2016-03-30]

41 Jannot AS, Vuillemin X and Etienne I et al. (2016) A Lack of Significant Effect of POR*28 Allelic Variant on Tacrolimus Exposure in Kidney Transplant Recipients. **Therapeutic Drug Monitoring**, 38 (2): 223-9

42 Elens L, Hesselink DA and Bouamar R et al. (2014) Impact of POR*28 on the pharmacokinetics of tacrolimus and cyclosporine A in renal transplant patients. **Therapeutic Drug Monitoring**, 36 (1): 71-9

43 Pulk RA, Schladt DS and Oetting WS et al. (2015) Multigene predictors of tacrolimus exposure in kidney transplant recipients. **Pharmacogenomics**, 16 (8): 841-54

44 Kuypers DR, de Loor H and Naesens M et al. (2014) Combined effects of CYP3A5*1, POR*28, and CYP3A4*22 single nucleotide polymorphisms on early concentration-controlled tacrolimus exposure in de-novo renal recipients. **Pharmacogenetics and genomics**, 24 (12): 597-606

45 Lunde I, Bremer S and Midtvedt K et al. (2014) The influence of CYP3A, PPARA, and POR genetic variants on the pharmacokinetics of tacrolimus and cyclosporine in renal transplant recipients. **European journal of clinical pharmacology**, 70 (6): 685-93

46 de Jonge H, Metalidis C and Naesens M et al. (2011) The P450 oxidoreductase *28 SNP is associated with low initial tacrolimus exposure and increased dose requirements in CYP3A5-expressing renal recipients. **Pharmacogenomics**, 12 (9): 1281-91

47 DiPiero J, Teng K and Hicks JK (2015) Should thiopurine methyltransferase (TPMT) activity be determined before prescribing azathioprine, mercaptopurine, or thioguanine?. **Cleveland Clinic Journal of Medicine**, 82 (7): 409-13

48 Linköping University IMH - Institutionen för medicin och hälsa **TPMT allele nomenclature** [online]. Available from: http://www.imh.liu.se/tpmtalleles/tabell-over-tpmt-alleler?l=en [Accessed 2016-05-09]

49 Daly AK and Arranz M (2012) "Pharmacogenetics of drug targets". In: Maitland-van der Zee A-H and Daly AK (eds.) **Pharmacogenetics and individualized therapy**. Hoboken: Wiley. pp. 149-182

50 Johnson JA, Gong L and Whirl-Carrillo M et al. (2011) Clinical Pharmacogenetics Implementation Consortium Guidelines for CYP2C9 and VKORC1 Genotypes and Warfarin Dosing [review]. **Clinical Pharmacology and Therapeutics**, 90 (4): 625-629

51 Verhoef TI, Redekop WK and Daly AK et al. (2014) Pharmacogenetic-guided dosing of coumarin anticoagulants: algorithms for warfarin, acenocoumarol and phenprocoumon [review]. **British Journal of Clinical Pharmacology**, 77 (4): 626-41

52 Takeuchi F, McGinnis R and Bourgeois S et al. (2009) A genome-wide association study confirms VKORC1, CYP2C9, and CYP4F2 as principal genetic determinants of warfarin dose. **PLoS Genetics**, 5 (3): e1000433

53 Ufer M, Svensson JO and Krausz KW et al. (2004) Identification of cytochromes P450 2C9 and 3A4 as the major catalysts of phenprocoumon hydroxylation in vitro. **European Journal of Clinical Pharmacology**, 60 (3): 173-82

54 Becquemont L (2008) Evidence for a pharmacogenetic adapted dose of oral anticoagulant in routine medical practice [review]. **European Journal of Clinical Pharmacology**, 64 (10): 953-60

 © 2014 - 2021 INTLAB AG

Laboratory

Laboratory analysis was carried out under Swiss law (GUMG) by:

labormedizinisches zentrum Dr Risch AG
Waldeggstrasse 37
CH-3097 Liebefeld
Telephone: +41 58 523 34 60
E-mail: genetik@risch.ch
Website: http://www.risch.ch

Version

Software: 1.9.0-0

Manufacturer

This report was generated by SONOGEN XP, an *in vitro* diagnostic medical device, manufactured by:

INTLAB AG
Seefeldstrasse 214
CH-8008 Zürich
Telephone: +41 43 508 69 36
E-mail: support@sonogen.eu
Website: http://www.sonogen.eu

© 2014 - 2021 INTLAB AG
